# Ribozyme-Mediated Downregulation Uncovers DNA Integrity Scanning Protein A (DisA) as a Solventogenesis Determinant in *Clostridium beijerinckii*

**DOI:** 10.3389/fbioe.2021.669462

**Published:** 2021-06-08

**Authors:** Victor Chinomso Ujor, Lien B. Lai, Christopher Chukwudi Okonkwo, Venkat Gopalan, Thaddeus Chukwuemeka Ezeji

**Affiliations:** ^1^Fermentation Science Program, Department of Food Science, University of Wisconsin-Madison, Madison WI, United States; ^2^Department of Chemistry and Biochemistry, Center for RNA Biology, The Ohio State University, Columbus, OH, United States; ^3^Department of Animal Sciences, Ohio State Agricultural Research and Development Center, The Ohio State University, Wooster, OH, United States

**Keywords:** butanol, carbon catabolite repression, M1GS, solventogenesis, metabolic engineering, *Clostridium beijerinckii*, RNase P

## Abstract

Carbon catabolite repression (CCR) limits microbial utilization of lignocellulose-derived pentoses. To relieve CCR in *Clostridium beijerinckii* NCIMB 8052, we sought to downregulate catabolite control protein A (CcpA) using the M1GS ribozyme technology. A CcpA-specific ribozyme was constructed by tethering the catalytic subunit of *Escherichia coli* RNase P (M1 RNA) to a guide sequence (GS) targeting CcpA mRNA (M1GS^CcpA^). As negative controls, the ribozyme M1GS^CcpA–Sc^ (constructed with a scrambled GS^CcpA^) or the empty plasmid pMTL500E were used. With a ∼3-fold knockdown of CcpA mRNA in *C. beijerinckii* expressing M1GS^CcpA^ (*C. beijerinckii*_M1GS^CcpA^) relative to both controls, a modest enhancement in mixed-sugar utilization and solvent production was achieved. Unexpectedly, *C. beijerinckii*_M1GS^CcpA–Sc^ produced 50% more solvent than *C. beijerinckii*_pMTL500E grown on glucose + arabinose. Sequence complementarity (albeit suboptimal) suggested that M1GS^CcpA–Sc^ could target the mRNA encoding DNA integrity scanning protein A (DisA), an expectation that was confirmed by a 53-fold knockdown in DisA mRNA levels. Therefore, M1GS^CcpA–Sc^ was renamed M1GS^DisA^. Compared to *C. beijerinckii*_M1GS^CcpA^ and _pMTL500E, *C. beijerinckii*_M1GS^DisA^ exhibited a 7-fold decrease in the intracellular c-di-AMP level after 24 h of growth and a near-complete loss of viability upon exposure to DNA-damaging antibiotics. Alterations in c-di-AMP-mediated signaling and cell cycling likely culminate in a sporulation delay and the solvent production gains observed in *C. beijerinckii*_M1GS^DisA^. Successful knockdown of the CcpA and DisA mRNAs demonstrate the feasibility of using M1GS technology as a metabolic engineering tool for increasing butanol production in *C. beijerinckii*.

## Introduction

The ability of solventogenic *Clostridium* species to utilize a wide range of sugar substrates to produce acetone, butanol, and ethanol (ABE) makes them suitable candidates for generating transport fuels and chemical feedstocks from renewable biomaterials (Jones and Woods, 1986; Grupe and Gottschalk, 1992; Ezeji et al., 2010). Among solvents produced by these Gram-positive, obligately anaerobic, spore-forming bacteria, butanol has drawn the most attention. Owing to its physico-chemical properties, butanol is an ideal transport fuel and has numerous applications in food, plastic, and rubber industries (Ezeji et al., 2003, 2010). However, bio-butanol production is currently economically unviable due to the high cost of substrates, low productivity, and low yield (Ezeji et al., 2007).

Lignocellulose, the most plentiful renewable resource for production of fermentable sugars, has been explored as an inexpensive substrate (Ho et al., 1998; Ezeji et al., 2007; Ren et al., 2010). Hydrolysis of lignocellulose releases mainly glucose and xylose with small amounts of other sugars including arabinose and mannose (Ho et al., 1998; Zverlov et al., 2006; Ezeji et al., 2007). Depending on the source, lignocellulosic biomass hydrolysates (LBH) can contain up to 58% glucose and 36% pentose (xylose + arabinose). While clostridia can utilize all these sugars, pentose utilization is drastically limited in the presence of glucose, an attribute that hampers solvent yield and productivity from LBH (Ounine et al., 1985; Mitchell, 1998; Ren et al., 2010). Thus, overriding this hierarchy in sugar utilization, a phenomenon named carbon catabolite repression (CCR), has been an important research objective in this field.

CCR is globally mediated by catabolite control protein A (CcpA), which in the presence of glucose represses the expression of numerous genes involved in the utilization of non-glucose substrates (Ludwig et al., 2002; Singh et al., 2008; Ren et al., 2010). Indeed, CcpA knockout in *Clostridium acetobutylicum* ATCC 824 led to concomitant utilization of glucose and xylose (Ren et al., 2010). However, compared to the wild type, this knockout strain was (i) less stable, (ii) produced less ABE and more acids (likely due to poor acid re-assimilation), and (iii) exhibited impaired sporulation (Ren et al., 2010, 2012). Such a broad range of effects was perhaps to be expected since CcpA, besides regulating carbon metabolism, exerts a pleiotropic effect on the expression of diverse genes unrelated to carbon utilization in *C. acetobutylicum* (Ren et al., 2012), *Bacillus subtilis* (Ludwig et al., 2002), *Enterococcus faecalis* (Leboeuf et al., 2000), and *Lactobacillus plantarum* (Mazzeo et al., 2012). In *C. acetobutylicum*, global transcriptomic analysis of the CcpA-null mutant relative to the wild type revealed that CcpA upregulates key solventogenic and sporulation genes, while negatively influencing the expression of acidogenic genes (Ren et al., 2012). Given the multiple roles of CcpA, we reasoned that a knockdown strain with some residual function would relieve CCR without engendering all the negative effects observed with the CcpA knockout. We therefore explored a ribozyme (RNase P)-mediated approach to knock down CcpA mRNA in *C. beijerinckii* NCIMB 8052.

**FIGURE 1:**
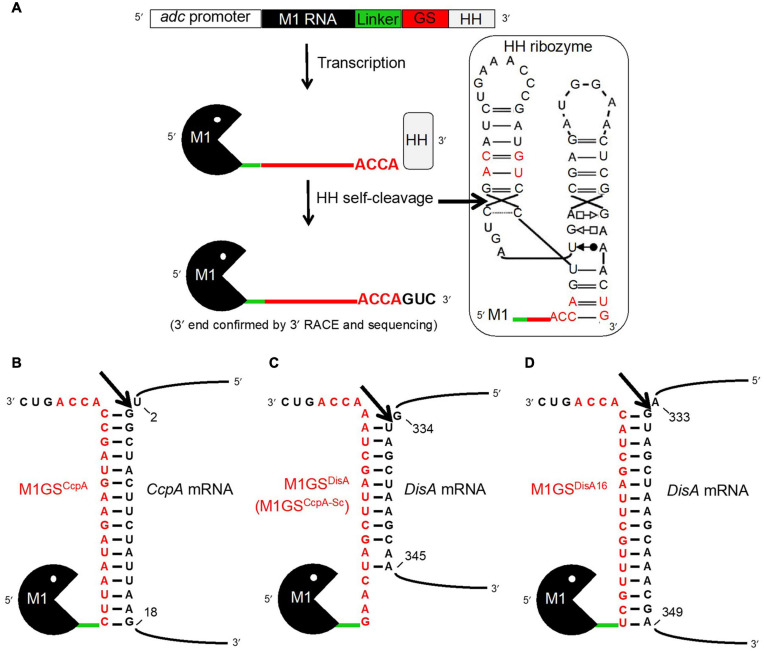
The M1GS approach. **(A)** The M1GS construct was transcriptionally controlled by the *C. acetobutylicum adc* promoter. A 51-nucleotide linker between M1 RNA and the GS was engineered to allow flexibility between them. After transcription, the hammerhead (HH) ribozyme self-cleaved with high efficiency at the precise location indicated by the arrow in the inset, leaving the M1GS transcripts with only three additional 3′ nucleotides. The inset shows the M1GS transcript with the full secondary structure of the HH ribozyme; the red-colored nucleotides are those that were modified to facilitate cloning and to minimize the number of additional bases at the 3′ ends of M1GS. Annealing of M1GS^CcpA^ **(B)**, M1GS^DisA^ **(C)**, and M1GS^DisA16^ **(D)** to their respective target mRNAs. The numbers indicate the nucleotide positions targeted by the ribozymes in the mRNAs (with AUG numbering 1-3). The arrows indicate where the tethered M1 RNA is expected to cleave the mRNAs.

In all domains of life, the primary function of RNase P is to remove the 5′ leader from precursor tRNAs (pre-tRNAs; Scott and Engelke, 2006; Altman, 2007; Lai et al., 2010). The ribonucleoprotein (RNP) form of bacterial RNase P comprises a catalytic RNA subunit (termed M1 RNA in *Escherichia coli*) and one protein cofactor. Any cellular RNA could be targeted for cleavage by RNase P (Forster and Altman, 1990; Guerrier-Takada and Altman, 2000) if the binding of the target RNA to a guide sequence (GS) forms a sequence- and structure-specific complex resembling the acceptor–T-stem helical stack in the pre-tRNA, a key recognition determinant of RNase P. The design also includes an (A/G)CCA sequence at the 3′ end of the GS to mimic the 3′ end of pre-tRNAs. An important variation that enhanced the efficiency of this method is the covalent tethering of M1 RNA to the GS (Li and Altman, 1996; Liu, 2010). When the GS in such an engineered M1GS binds an accessible, single-stranded region in its target RNA to form a stem substrate, the bipartite substrate is cleaved *in cis* by the covalently attached M1 RNA (Figure 1A). Scrambling the GS while retaining the nucleotide composition provides a control to assess specificity of targeting. Given the proven utility of this method for targeted degradation of RNAs in bacteria, mammalian cells, and mice (Li and Altman, 1996; Bai et al., 2010, 2011; Liu, 2010), we sought its application for knocking down CcpA in *C. beijerinckii*.

While expression of a customized CcpA-specific M1 RNA-based ribozyme (M1GS^CcpA^) in *C. beijerinckii* displayed a 3-fold knockdown in the CcpA mRNA level and a small but discernible increase in ABE production, we unexpectedly found that the specificity control M1GS with a scrambled GS^CcpA^ resulted in a 1.5-fold increase in ABE titer compared to a strain transformed with the empty vector. Upon further investigation, we found that the scrambled GS^CcpA^ targeted the mRNA encoding DNA integrity scanning protein A (DisA) and caused a 53-fold decrease in the DisA mRNA steady-state level. DisA monitors genomic integrity at the onset of sporulation and recruits the DNA repair machinery upon detection of DNA damage (Oppenheimer-Shaanan et al., 2011). While DisA scans the genomic DNA, it produces cyclic diadenosine monophosphate (c-di-AMP), a second messenger critical for cellular homeostasis (Stülke and Krüger, 2020). In fact, c-di-AMP activates Spo0A, a master transcriptional regulator of sporulation. Thus, M1GS^DisA^-mediated knockdown of DisA might enhance solventogenesis by delaying sporulation. In addition to uncovering this unanticipated nexus between DisA, c-di-AMP, and ABE production, our work demonstrates the utility of the M1GS technology as a tool for metabolic engineering of *C. beijerinckii* and likely other solventogenic *Clostridium* species.

## Materials and Methods

### M1GS Design and Cloning

Translational initiation requires binding of the ribosome to a single-stranded region near the start codon. Therefore, we used the mfold web server (Zuker, 2003), which predicts RNA secondary structures based on energy minimization, to determine single-strandedness in nucleotides –25 to +55 of the CcpA open reading frame (ORF). Such an analysis led us to design GS^CcpA^ for targeting nucleotides 3-18 of the CcpA ORF (Figure 1B). The scrambled version GS^CcpA–Sc^, which was originally intended as a negative control for GS^CcpA^, was subsequently discovered to fortuitously target nucleotides 335-343 of the DisA ORF (Figure 1C). These sequences including the 3′ACCA were inserted between the linker and the hammerhead (HH) ribozyme sequences in pBT7–M1-HH, a template vector that harbored sequences for M1 RNA, a 51-nucleotide linker, and a HH ribozyme in tandem (Figure 1A) and downstream of the T7 RNA polymerase promoter. Using inverse PCR and back-to-back primers (Table 1) flanking the linker-HH border [GS/HH-F and a reverse primer that includes the GS sequence: GS(CcpA)-R or GS(CcpA-Sc)-R], the entire pBT7–M1GS-HH plasmid was amplified, circularized by ligation, and used to transform *E. coli* DH5α cells. Subsequently, primers 5′M1(*Apa*I)-F and 3′HH(*Xho*I)-R were used to introduce an *Apa*I and a *Xho*I site by PCR just upstream of M1 RNA and downstream of HH, respectively, using pBT7–M1GS-HH as the template. After digestion with *Apa*I and *Xho*I, the PCR products were cloned into pWUR459 (under the control of an inducible *adc* promoter; Siemerink et al., 2011) that had been digested with the same restriction enzymes.

**TABLE 1:** Sequences of oligonucleotides used in this study.

**Primer Name**	**Target gene/template**	**Sequence (5**′→ **3′)**
GS/HH-F	M1GS	ACCAGTCGACA TCTGAAACCC
GS(CcpA)-R	M1GS^CcpA^	GGCTACTTCTATT AAGAAACCTATG ACCATGATTA
GS(CcpA-Sc)-R	M1GS^DisA^	TTAGCTAAGCTAGT TCAAACCTATGA CCATGATTA
5′M1(*Apa*I)-F	M1GS	CATGGGCCC GAAGCTGACCAGAC
3′HH(*Xho*I)-R	M1GS	CGTGCTCGAGG TGAAACTGACC
M1-F	*E. coli* M1 RNA	GACCAGTGC AACAGAGAGC
M1-R	*E. coli* M1 RNA	GTCGTGGACA GTCATTCATC
Cbei_R0124-F	*C. beijerinckii* 16S rRNA	GAAGAATACCAG TGGCGAAGGC
Cbei_R0124-R	*C. beijerinckii* 16S rRNA	ATTCATCGTT TACGGCGTGGAC
Cbei_0047-F	*C. beijerinckii* CcpA	GTTGCAAAAG AAGCAGGAG
Cbei_0047-R	*C. beijerinckii* CcpA	CAGCACCTCT TACAATTTCTG
Cbei_0127-F	*C. beijerinckii* DisA	GAAACAGGAAC TAGGCATAGAAC
Cbei_0127-R	*C. beijerinckii* DisA	CTTGATTTGCCT TTCCAAG
M1p3-F (delta-M1GS)	*C. beijerinckii* DisA-16	GCTTCGTCGTC GTCCTCTTCG
M1p12-R (delta-M1GS)	*C. beijerinckii* DisA-16	CCATCGGCGG TTTGCTCTCTG
F-ext	pBT7 vector	CGACGTTGTAAAA CGACGGCCAG
M1GS-3F	M1GS	GAAGCTGACCA GACAGTCGC
M1-4F	M1GS	AGGGTGCCA GGTAACGCC

Since only nine contiguous nucleotides in DisA ORF were complementary to M1GS^DisA^ (Figure 1C), we constructed M1GS^DisA16^ that targets 16 nucleotides (334-349) in DisA ORF (Figure 1D). For use as a negative control, we generated ΔM1GS^DisA16^ where an M1 RNA deletion mutant (ΔM1) substituted for the wild type such a control provides an opportunity to assess gene expression changes arising solely from antisense effects (Liu and Altman, 1995; Gopalan et al., 2002). All the cloned inserts were verified by sequencing before using to transform *C*. *beijerinckii.*

### Strains and Culture Conditions

*Clostridium beijerinckii* NCIMB 8052 was obtained from the American Type Culture Collection (Manassas, VA, United States) as *C*. *beijerinckii* ATCC 51743 (the same strain is labeled differently as NCIMB 8052 by the NCIMB culture collection, United Kingdom). Laboratory stocks were maintained as spore suspensions in sterile, double-distilled water at 4°C. All cultures were grown at 35 ± 1°C in tryptone-glucose-yeast extract (TGY; 30, 20, and 10 g/L, respectively) broth, and in an anaerobic chamber (Coy Laboratory Products Inc., Grass Lake, MI, United States) with a modified atmosphere of 82% N_2_, 15% CO_2_, and 3% H_2_.

To obtain *C*. *beijerinckii*_M1GS^DisA^, *_*M1GS^CcpA^, _M1GS^DisA16^, ΔM1GS^DisA16^, and _pMTL500E, transformation of *C*. *beijerinckii* with the corresponding M1GS constructs or the parental plasmid pMTL500E was conducted as described by Kim and Blaschek (1993) with minor modifications. First, a starter culture was grown for 12 h before sub-culturing [10% (v/v) inoculum] into fresh TGY broth and grown until late-exponential phase (OD_600_ ∼1.2). For each transformation, 10 mL of cells were harvested, washed with electroporation solution [10% (w/v) polyethylene glycol 8000 in distilled water], and re-suspended in 1 mL of the same solution. Four hundred μl of this suspension was then mixed with 10 μg of plasmid DNA and subjected to electroporation at 2.5 kV, 25 μF, and infinite resistance in a Gene Pulser (Bio-Rad, Hercules, CA, United States). Subsequently, cells were quickly transferred to 5 mL of TGY broth and grown for 8 h, before being plated on TGY agar [0.45% (w/v) agar] containing erythromycin (25 μg/mL) and incubated overnight at 35 ± 1°C. Single colonies were then suspended in 500 μl of TGY, re-streaked on TGY agar (+25 μg/mL erythromycin), and incubated overnight at 35 ± 1°C. Fresh colonies were then picked and grown in TGY broth for 12 h to make a glycerol stock. In parallel, cells were sub-cultured into P2 medium containing 60 g/L glucose (+25 μg/mL erythromycin) and grown for 7 days to generate spores for subsequent experiments. The P2 medium (100 mL) used for fermentative characterization of the strains contained 60 g/L glucose, arabinose or xylose, or glucose-pentose mixtures totaling 60 g/L sugars [40 g/L glucose and 20 g/L pentose (arabinose or xylose)] and 1 g/L yeast extract supplemented with 1 mL each of buffer, vitamin, and mineral stocks. The buffer stock contained K_2_HPO_4_ (50 g/L) and ammonium acetate (220 g/L), while the vitamin stock contained *p*-amino-benzoic acid (0.1 g/L), thiamine (0.1 g/L), and biotin (0.001 g/L). The mineral stock comprised MgSO_4_⋅7H_2_O (20 g/L) MnSO_4_⋅H_2_O (1 g/L), FeSO_4_⋅7H_2_O (1 g/L), and NaCl (1 g/L).

For fermentation, all cultures were grown in loosely capped 250-mL Pyrex culture bottles at 35 ± 1°C. For phenotypic characterization of *C*. *beijerinckii*_M1GS^DisA^, *_*M1GS^CcpA^, _M1GS^DisA16^, _ΔM1GS^DisA16^, and _pMTL500E, inoculum generation and fermentation were conducted as described above (also see Ujor et al., 2014). Cultures for the determination of intracellular c-di-AMP levels were grown in the glucose + arabinose medium for 60 h with aliquots withdrawn every 12 h starting at 0 h. In addition to the standard potassium phosphate buffer (K_2_HPO_4_, 0.5 g/L final concentration), all fermentation media were buffered with 2-(N-morpholino) ethanesulfonic acid (MES; 7 g/L) and supplemented with erythromycin (25 μg/mL).

### RNA Isolation and RT-Quantitative PCR (RT-qPCR)

Cell pellets from 6 mL of culture grown in glucose + arabinose medium for 24 h were used for RNA isolation. RNA was isolated individually from triplicate cultures of *C*. *beijerinckii* strains (_M1GS^DisA^, _M1GS^CcpA^, _M1GS^DisA16^, _ΔM1GS^DisA16^, or _pMTL500E). Each cell pellet was suspended in 1 mL of TRI Reagent (Sigma, St. Louis, MO, United States) and lysed by passing through Tissue Lyser LT (Qiagen, Hilden, Germany) at maximal oscillation for 2 min. Subsequent RNA isolation steps were performed as per manufacturer’s instructions. Reverse transcription of RNA was carried out with 2 μg of total RNA using random hexamers and M-MLV Reverse Transcriptase (Promega, Madison, WI, United States). Quantitative PCR (20 μl final volume) was then conducted with DyNAmo HS SYBR Green qPCR Master Mix (Thermo Fisher Scientific, Waltham, MA, United States), using gene-specific primers (Table 1) for M1 RNA (M1-F & R), ΔM1RNA (M1p3-F & M1p12-R), CcpA (Cbei_0047- F & R), and DisA (Cbei_0127- F & R). Each of the triplicate RNA preparations was analyzed twice in a BioRad iCycler continuous fluorescence detection system (BioRad, Hercules, CA, United States), with the following cycling conditions: 95°C – 15 min; 40 cycles of 95°C – 10 s and 55°C – 60 s. The levels of respective RNAs were normalized to 16S rRNA (Zhang and Ezeji, 2013).

### Determination of M1GS and DisA Copy Number per Cell

The reference RNAs were first generated using run-off *in vitro* transcription. For M1GS, the reference M1 RNA was obtained using as template *Fok*I-linearized pJA2′ (Vioque et al., 1988). For the DisA mRNA, a fragment corresponding to nucleotides 301-460 of the DisA ORF was first amplified by PCR using primers Cbei_0127-F and Cbei_0127-R (Table 1) and cloned downstream of the T7 promoter in *Stu*I-digested pBT7 (Tsai et al., 2002). Following screening for a clone with an insert in the sense orientation and subsequent confirmation by sequencing, pBT7-DisA was used as the template for PCR with primers F-ext (Table 1) and Cbei_0127-R to generate the DNA template for *in vitro* transcription of the DisA RNA fragment. Subsequently, based on the molecular weight and the concentration (determined using a spectrophotometer), the copy number of each transcript was calculated as previously described (Devonshire et al., 2016).

Standard curves were generated by performing RT-qPCR with 10, 20, 30, 50, 80, and 100 copies of M1 RNA and DisA transcripts and with primer sets M1-F + M1-R and Cbei_0127-F + Cbei_0127-R, respectively. The resulting quantification cycles (Cq) were then plotted against the known copy numbers. RNA samples (2 μg) isolated from *C*. *beijerinckii*_M1GS^DisA^, _M1GS^CcpA^, or _pMTL500E were then analyzed by RT-qPCR in the same manner. Based upon the Cq values obtained for each reaction, the corresponding copy numbers for M1GS^DisA^ or M1GS^CcpA^ were interpolated from the respective standard curves and normalized by subtracting the background value obtained using RNA isolated from *C*. *beijerinckii*_pMTL500E. This background correction was motivated by the expectation that neither M1GS^DisA^ nor M1GS^CcpA^ should be present in *C*. *beijerinckii* transformed with pMTL500E. The Cq values obtained for M1GS and DisA transcripts were within the range of the standard curve. By plating cells on TGY agar (as described above), we counted the colonies from cells harvested for RNA extraction and calculated the number of cells/culture volume. With this information as well as the RT-qPCR results, we could determine the number of M1GS and DisA copies per cell for the strains in this study.

### Rapid Amplification of cDNA 3′ Ends (3′ RACE) Analysis

To assess the efficacy of the HH ribozyme cleavage and generation of the intended M1GS RNA, we sought to map the 3′ termini of the M1GS ribozymes from *C*. *beijerinckii*_M1GS^DisA^ and _M1GS^CcpA^. To this end, total RNA was first incubated with polynucleotide kinase (37°C – 2 h) to dephosphorylate the 2′,3′-cyclic phosphate produced by HH cleavage. The RNA was then polyadenylated using the poly(A) Tailing Kit (Invitrogen, Carlsbad, CA, United States). Subsequently, the RNA was reverse transcribed using SuperScript II (Invitrogen) and the anchored oligo dT primer from the GeneRacer Kit (Invitrogen). The resulting cDNAs were then treated with RNase H before amplifying by PCR the 3′ ends of the ribozymes with M1GS-3F and the GeneRacer 3′ Primer using PrimeSTAR GXL (Takara Bio USA, Mountain View, CA, United States). This step was followed by a round of nested PCR using M1-4F and the GeneRacer 3′ Nested Primer. The nested-PCR RACE product, which migrated close to the expected size on an agarose gel, was then sequenced with the M1-4F primer at the OSU Genomics Shared Resources facility. Sequencing results showed that the HH ribozyme cleaved at the expected position and generated the desired 3′ termini (data not shown).

### Analytical Methods

Intracellular levels of c-di-AMP were analyzed by HPLC using the Waters 2796 Bioseparations Module (Waters Corporation, Milford, MA, United States), equipped with a photodiode array (PDA) detector (Waters Corporation, Milford, MA, United States) and a 3.5-μm Xbridge C18, 150 mm × 4.6 mm column (Waters Corporation, Milford, MA, United States) according to Oppenheimer-Shaanan et al. (2011). Pure c-di-AMP (BIOLOG Life Science Institute, Bremen, Germany) was used as standard. C-di-AMP was extracted from cell pellets according to Oppenheimer-Shaanan et al. (2011). Pellets were obtained from triplicate 50-mL samples taken every 12 h from cultures grown in glucose + arabinose medium, and each extract was analyzed twice. Extracts were reconstituted in sterile distilled water (400 μl) for HPLC analysis.

Acetone, butanol, ethanol, acetic acid, and butyric acid concentrations were quantitated using a 7890A Agilent gas chromatograph (Agilent Technologies Inc., Santa Clara, CA, United States) as described previously (Ujor et al., 2014). The GC data were analyzed using Agilent Chem Station software (Rev. B.03.02 SR2). Cell growth was determined by measuring optical density (OD_600_) using a DU^®^ spectrophotometer (Beckman Coulter, Brea, CA, United States). The concentrations of glucose, arabinose and xylose were determined by HPLC using the Waters 2796 Bioseparations Module (Waters Corporation, Milford, MA, United States) as previously described (Ujor et al., 2014).

### Mitomycin C and Nalidixic Acid Sensitivity Assays

At 25 h post inoculation into glucose + arabinose medium, triplicate cultures of *C*. *beijerinckii*_M1GS^DisA^, _M1GS^CcpA^ or _pMTL500E were adjusted to the same optical density (OD_600_), and then diluted 1:500 (in 1 mL) before plating out on semi-solid TGY agar [0.45% (w/v) agar] containing erythromycin (20 ng/mL) and mitomycin C (50, 80, or 120 ng/mL) or nalidixic acid (470, 480, or 495 ng/mL). Plates were incubated for 14 to 24 h at 35°C ± 1°C prior to counting the colony forming units.

### Sporulation Test

To determine the progression of sporulation in cultures of *C*. *beijerinckii*_M1GS^DisA^, _M1GS^CcpA^, or _pMTL500E, all three strains were grown in glucose + arabinose medium in triplicate. Samples were taken from each culture every 12 h and diluted 1:100 (in 1 mL). A portion of the sample (500 μl) was heat-shocked at 75°C for 8 min, cooled on ice and plated on TGY agar to determine the number of spores per sample. The remainder of each sample was plated out without heat treatment to determine the number of vegetative cells in the samples relative to the spores (heat-shocked portion). All plates contained erythromycin (25 μg/mL) and were incubated for 24 h as described above, and the colonies were counted.

### Statistical Analysis

Statistical analyses were conducted with the General Linear Model (GLM) of Minitab version 17 (Minitab Inc., State College, PA, United States). Statistical analyses compared the differences in the results obtained with *C*. *beijerinckii*_M1GS^DisA^, _M1GS^CcpA^, _M1GS^DisA16^, or _pMTL500E including butanol and ABE concentrations, yields and productivities, cell growth, c-di-AMP levels, copy numbers and fold changes in M1GS and DisA mRNA levels, and residual sugar concentrations after fermentation with each strain. Data were un-stacked by time and ANOVA was conducted at different time points. Tukey’s test at 95% confidence interval was applied to pairwise comparisons to determine the levels of significance. All experiments were performed in triplicate, and the errors reported reflect the standard deviation of the calculated mean.

## Results

### M1GS Design and Construction

The M1GS approach is depicted in Figure 1A. Before designing an M1GS construct against an mRNA, it is necessary to identify a single-stranded region (preferably proximal to the start codon, A_+__1_UG) in the target mRNA. Analysis of the CcpA mRNA secondary structure [nucleotides –25 to +55 of the ORF] using the mfold web server (Zuker, 2003) indicated that nucleotides +2 to +18 of its ORF are likely to be accessible for base pairing to a complementary GS. We then constructed the genes encoding two customized ribozymes: one was the test M1GS^CcpA^ and the other a scrambled control M1GS^CcpA–Sc^. The GS^CcpA–Sc^ has the same nucleotide composition as GS^CcpA^ but a different sequence (Figures 1B,C); we ascertained by BLAST that the scrambled GS sequence did not have a perfect (16-nucleotide) contiguous match with any other mRNA in the *C. beijerinckii* genome.

To ensure a uniform 3′ terminus for each M1GS transcript generated *in vivo*, we placed a HH ribozyme downstream of M1GS. Self-cleaving HH ribozymes have been used to synthesize RNAs with well-defined termini *in vitro* and *in vivo*. Both M1GS^CcpA^ and M1GS^CcpA–Sc^ constructs (Figures 1B,C) were cloned into pWUR459 (Siemerink et al., 2011), displacing the resident *acetoin reductase* gene, and placing the constructs under the control of the acetoacetate decarboxylase (*adc*) promoter, which is active during solventogenesis. As an additional negative control, we used pMTL500E, the vector from which pWUR459 originated. All three plasmids were introduced into *C. beijerinckii* by electroporation to generate the strains *C. beijerinckii*_M1GS^CcpA^, _M1GS^CcpA–Sc^, and _pMTL500E.

Before examining the effect of CcpA knockdown on solventogenesis, we determined that M1GS ribozymes were expressed in *C. beijerinckii*_M1GS^CcpA^ and _M1GS^CcpA–Sc^ relative to the vector control strain *C. beijerinckii*_pMTL500E (Table 2), and that they were synthesized with the correct termini. Although some earlier reports used only a minimal HH catalytic core (Uhlenbeck, 1987), since loop sequences outside the catalytic core were found to enhance the rate of self-cleavage at physiological Mg^2+^ concentrations (Khvorova et al., 2003), we opted for a naturally occurring, loop-containing HH ribozyme found in *Arabidopsis thaliana* (Ara1, k_obs_ ∼2 min^–1^ at 25°C in 0.6 mM Mg^2+^) (Przybilski et al., 2005) with some modifications (Figure 1A, inset). Indeed, 3′ RACE followed by sequencing proved that Ara1, albeit of plant origin, functions efficiently in *C. beijerinckii* and cleaves at the expected position to generate a precise 3′ terminus (data not shown). To our knowledge, such use of HHs in the M1GS approach has not been reported.

**TABLE 2:** Determination of fold-change for CcpA and DisA mRNA level and of the cellular copy number for M1GS and DisA transcripts.

***C. beijerinckii* strain**	**CcpA mRNA (fold change)**	**DisA mRNA (fold change)**	**M1GS transcript (copies/cell)**	**DisA mRNA (copies/cell)**
pMTL500E	1.00.0	1.00.0	0.00.0	791.078.0
M1GS^CcpA^	−2.80.4	1.00.0	839	828.074.0
M1GS^DisA^	1.00.0	−44.04.4	878	15.32.8
MIGS^DisA16^	1.00.0	−43.51.0	ND	ND
ΔMIGS^DisA16^	1.00.0	−1.50.0	ND	ND

*ND, not determined. Tukey’s pairwise comparison was applied to the means of CcpA mRNA fold changes, M1GS transcript copies, and DisA mRNA copies. RNA copy number was calculated using RT-qPCR (see text for details). All fold changes are relative to *C. beijerinckii*_pMTL500E. Errors denote the standard deviation of the mean (*n* = 3).*

### M1GS^CcpA^ Elicited Knockdown of CcpA mRNA and a Modest Increase in ABE Production

**FIGURE 2:**
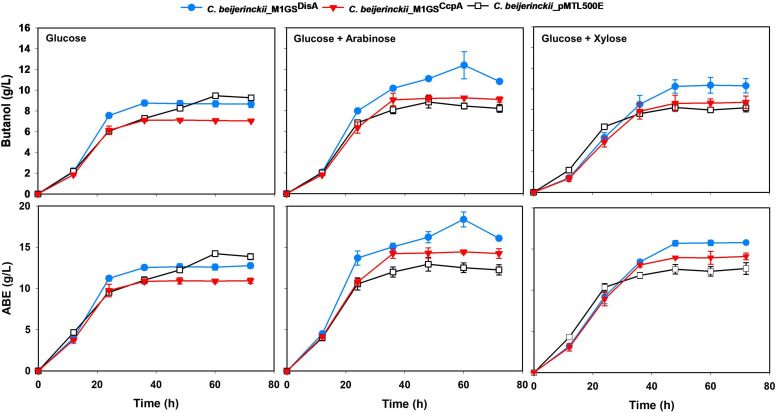
Butanol and ABE titers during a 72-h fermentation in cultures of *C. beijerinckii*_pMTL500E, _M1GS^CcpA^, and _M1GS^DisA^ on glucose, glucose + arabinose, and glucose + xylose. Error bars represent standard deviation of the mean (*n* = 3).

We used RT-qPCR with CcpA-specific primers (Table 1) that flank the targeted region to determine the expression of CcpA mRNA in the three *C. beijerinckii* transformants. We found that the CcpA mRNA level in *C. beijerinckii_*M1GS^CcpA^ decreased 2.8-fold (*p* < 0.05) when compared to the other strains (Table 2). While examining the ABE production profiles for all strains grown on various sugars, the largest increase observed in *C. beijerinckii*_M1GS^CcpA^ relative to _pMTL500E was ∼1.2-fold (*p* < 0.05) when grown on glucose + arabinose (Figure 2 and Table 3). Unexpectedly, we also observed a ∼1.5-fold (*p* < 0.05). Increase in ABE production by *C. beijerinckii*_M1GS^CcpA–Sc^ compared to the vector control, both grown on glucose + arabinose (Figure 2 and Table 3). We leveraged this adventitious finding to direct our study differently.

**TABLE 3:** Maximal butanol **(A)** and ABE **(B)** levels in cultures of different *C. beijerinckii* strains grown on different sugars for 60 h.

**(A)**										
**Butanol**	**Glucose medium**		**Glucose + Arabinose medium**		**Glucose + Xylose medium**		**Arabinose medium**		**Xylose medium**	
***C. beijerinckii* strain**	**Conc. (g/L)**	*Fold change	**Conc. (g/L)**	*Fold change	**Conc. (g/L)**	*Fold change	**Conc. (g/L)**	*Fold change	**Conc. (g/L)**	*Fold change
pMTL500E	9.50.2	1.00.0^a^	8.50.4	1.00.0^a^	8.20.4	1.00.0^a^	10.20.1	1.00.0^a^	9.60.3	1.00.0^a^
M1GS^*CcpA*^	7.10.1	−1.30.1^c^	9.20.0	1.10.0^a^	8.70.6	1.10.1^a^	11.00.1	1.10.0^a^	9.60.7	1.00.0^a^
M1GS^*DisA*^	8.70.3	−1.10.1^a^	12.51.6	1.50.6^c^	10.40.8	1.30.4^c^	12.20.4	1.20.1^c^	10.20.8	1.10.2^a^
**(B)**										
**ABE**	**Glucose medium**		**Glucose + Arabinose medium**		**Glucose + Xylose medium**		**Arabinose medium**		**Xylose medium**	
***C. beijerinckii* strain**	**Conc. (g/L)**	*Fold change	**Conc. (g/L)**	*Fold change	**Conc. (g/L)**	*Fold change	**Conc. (g/L)**	*Fold change	**Conc. (g/L)**	*Fold change
pMTL500E	14.20.4	1.00.0^a^	12.50.6	1.00.0^a^	12.60.7	1.00.0^a^	14.00.2	1.00.0^a^	14.500.6	1.00.0^a^
M1GS^*CcpA*^	10.90.4	−1.30.1^c^	14.50.2	1.20.1^c^	14.10.4	1.10.1^c^	15.00.4	1.10.1^a^	14.51.5	1.00.8^a^
M1GS^*DisA*^	12.80.3	−1.10.1^c^	18.40.9	1.50.0^c^	15.80.2	1.30.0^c^	17.50.5	1.30.2^c^	15.60.6	1.10.2^a^

*All fold changes are relative to *C. beijerinckii*_pMTL500E. Tukey’s pairwise comparison was applied to the mean fold changes in butanol and ABE concentrations in cultures of *C. beijerinckii*_M1GS^CcpA^ and _M1GS^DisA^ relative to those of *C. beijerinckii*_pMTL500E. Fold changes for *C. beijerinckii*_pMTL500E, _M1GS^CcpA^, and _M1GS^DisA^ with different superscripts within a column are significant (*p* ≤ 0.05). Errors denote standard deviation of the mean (*n* = 3).

### M1GS^CcpA–Sc^ (M1GS^DisA^) Leads to a Pronounced Decrease in DisA mRNA Level

To understand the basis for increased ABE production by *C. beijerinckii*_M1GS^CcpA–Sc^, we performed a BLASTn analysis using the GS^CcpA–Sc^ sequence as a query against the *C. beijerinckii* genome. Although we found several genes with a complementarity of nine or more contiguous nucleotides, only six of them seemed likely to affect fermentation. When RT-qPCR analysis of these six mRNAs was conducted (Supplementary Table 1), only Cbei_0127 (DisA) mRNA was found to decrease drastically (44-fold; *p* < 0.05) in *C. beijerinckii*_M1GS^CcpA–Sc^ relative to *C. beijerinckii*_M1GS^CcpA^ and _pMTL500E. This finding prompted us to rename M1GS^CcpA–Sc^ as M1GS^DisA^.

In an independent RT-qPCR experiment, we used an *in vitro* transcribed fragment of the DisA mRNA as a reference for a standard curve and determined that ∼800 copies of DisA mRNA are present per cell in *C. beijerinckii*_M1GS^CcpA^ and _pMTL500E, but only 15 copies in *C. beijerinckii*_M1GS^DisA^, representing a 53-fold (*p* < 0.05) downregulation mediated by M1GS^DisA^ (Table 2). With *in vitro* transcribed M1 RNA as a reference for a standard curve, we found that the normalized copy number of M1GS per cell is 0, 83, and 87, in *C. beijerinckii*_pMTL500E, _M1GS^CcpA^, and _M1GS^DisA^, respectively (Table 2). The decrease from 828 to 15 copies of DisA mRNA mediated by 87 copies of M1GS^DisA^ in *C. beijerinckii*_M1GS^DisA^ must entail multiple turnover of the ribozyme (at least nine rounds). Although multiple turnover was an implicit expectation for M1GS, *in vivo* evidence has not been documented before.

### *C. beijerinckii*_M1GS^DisA^ Displayed a Decreased c-di-AMP Level, Increased Sensitivity to DNA-Damaging Antibiotics, and a Slower Sporulation Rate

**FIGURE 3:**
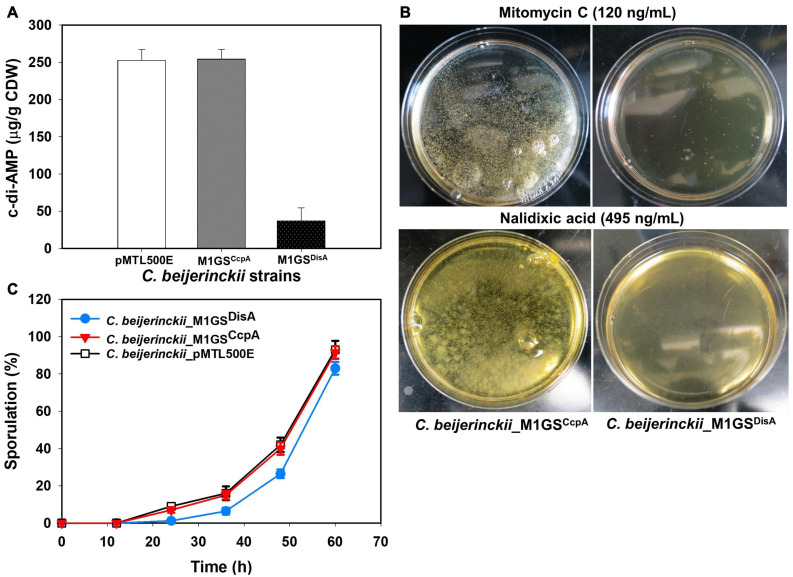
Phenotypic changes elicited by M1GS^DisA^. **(A)** Intracellular c-di-AMP levels in *C. beijerinckii*_M1GS^DisA^ decreased 7-fold relative to *C. beijerinckii* _M1GS^CcpA^ and _pMTL500E at 24 h of growth. CDW, cell dry weight. **(B)** *C. beijerinckii*_M1GS^DisA^ was more sensitive to mitomycin C and nalidixic acid than *C. beijerinckii*_M1GS^CcpA^. At the specified concentrations, both antibiotics caused ∼100% loss of cell viability in *C. beijerinckii*_M1GS^DisA^ (see Supplementary Figure 1 for more details). Generation of carbon dioxide and hydrogen during cellular growth and metabolism caused bubbles on *C. beijerinckii*_M1GS^CcpA^ plates. **(C)** Sporulation profiles of *C. beijerinckii*_M1GS^DisA^ relative to *C. beijerinckii*_M1GS^CcpA^ and _pMTL500E. Error bars represent standard deviation of the mean (*n* = 3).

Since DisA is a diadenylate cyclase (DAC) that synthesizes c-di-AMP (Bejerano-Sagie et al., 2006; Oppenheimer-Shaanan et al., 2011), its knockdown should result in decreased c-di-AMP production. Indeed, when intracellular levels of c-di-AMP in 24-h cultures of *C. beijerinckii*_M1GS^DisA^, _M1GS^CcpA^, and _pMTL500E were quantitated by HPLC, we observed a 7-fold (*p* < 0.05) decrease in *C. beijerinckii*_M1GS^DisA^ compared to the other two strains (Figure 3A).

Because DisA scans genomic DNA and initiates the repair of lesions, we also postulated that *C. beijerinckii*_M1GS^DisA^ would be more susceptible to DNA-damaging agents due to DisA knockdown. Therefore, *C. beijerinckii*_M1GS^DisA^ and _M1GS^CcpA^ were challenged with mitomycin C and nalidixic acid, two well-established DNA-damaging antibiotics. Both drugs drastically decreased the viability of *C. beijerinckii*_M1GS^DisA^ compared to _M1GS^CcpA^ (Figure 3B and Supplementary Figure 1). At 120 ng/mL of mitomycin C, the viability of M1GS^DisA^ decreased by 99.9% (*p* < 0.05). A similar effect was observed with nalidixic acid, which decreased the viability of *C. beijerinckii*_M1GS^DisA^ by 99.8% (*p* < 0.05) at 470 ng/mL and by 100% at 480-495 ng/mL. These results indicate that *C. beijerinckii*_M1GS^DisA^ is hypersensitive to DNA-damaging antibiotics.

DisA monitors genomic integrity particularly at the onset of sporulation. To allow a cell to fix damages to DNA that might disrupt its replication or cause defects in chromosome partitioning, DisA halts sporulation by delaying the activation of Spo0A, a master transcriptional activator critical for sporulation. Thus, DisA knockdown is expected to alter the sporulation dynamics in *C. beijerinckii*_M1GS^DisA^. Since cultures of solventogenic clostridia exist as mixtures of vegetative cells and spores upon transition to solventogenesis, we assessed the progression of sporulation in cultures of *C. beijerinckii*_M1GS^DisA^, _M1GS^CcpA^, and _pMTL500E over a 60-h fermentation (Figure 3C). As expected, *C. beijerinckii*_M1GS^DisA^ showed a delay relative to *C. beijerinckii*_pMTL500E (Figure 3C). We observed 7.4−, 2.5− and 1.6-fold decrease in percent sporulation at 24, 36, and 48 h, respectively, for *C. beijerinckii*_M1GS^DisA^ compared to *C. beijerinckii*_pMTL500E (*p* < 0.05). Although CcpA is known to partake in sporulation, no substantial delay was found in *C. beijerinckii*_M1GS^CcpA^ cultures compared to the sporulation rate found in *C. beijerinckii*_pMTL500E cultures. It is also possible that the ∼3-fold decrease in CcpA mRNA level is insufficient to cause a change in the sporulation dynamics.

### Both *C. beijerinckii*_M1GS^CcpA^ and _M1GS^DisA^ Exhibited Increased Solvent Production in Pentose-Containing Media

**FIGURE 4:**
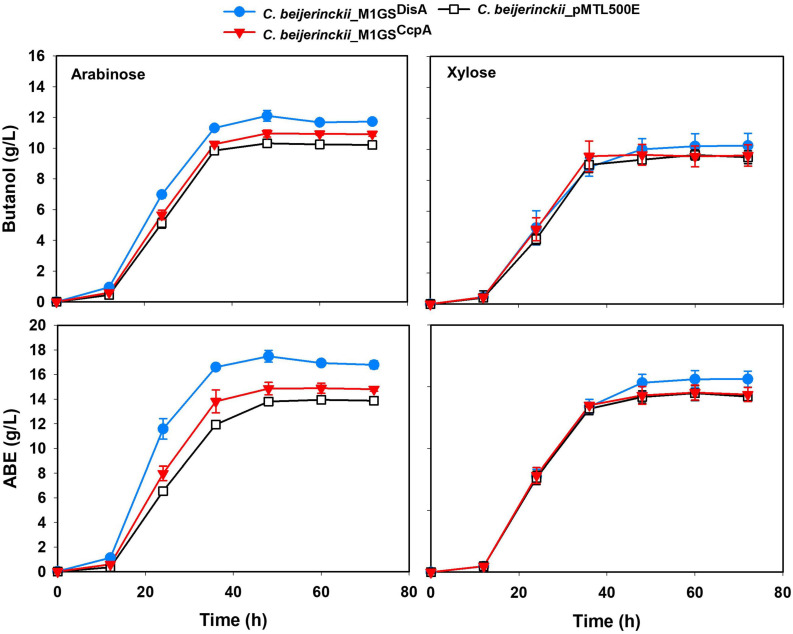
Butanol and ABE titers during a 72-h fermentation in cultures of *C. beijerinckii*_ pMTL500E, _M1GS^CcpA^, and _M1GS^DisA^ grown on arabinose and xylose. Error bars represent standard deviation of the mean (*n* = 3).

The effects of CcpA and DisA knockdown on butanol and ABE production were assessed while growing on media with single (glucose, arabinose, or xylose) and mixed sugars (glucose + arabinose or glucose + xylose) as carbon source(s). Interestingly, butanol production by both *C. beijerinckii*_M1GS^CcpA^ and _M1GS^DisA^ was lower than the control (*C. beijerinckii*_pMTL500E) when grown on glucose (Table 3), although *C. beijerinckii_*M1GS^DisA^ initially (12-48 h) produced more than the other two strains (Figure 2). Except for *C. beijerinckii*_M1GS^CcpA^ being on par with the control in xylose medium (Figure 4), *C. beijerinckii*_M1GS^CcpA^ and _M1GS^DisA^ showed higher butanol production than the control strain in all pentose-containing media (Figures 2, 4 and Table 3). This difference was most pronounced in media containing arabinose, either alone or in combination with glucose (Figures 2, 4). In the arabinose medium, maximal butanol concentration was 1.2-fold (*p* < 0.05) higher in *C. beijerinckii*_M1GS^DisA^ than *C. beijerinckii*_pMTL500E; remarkably, this change increased to 1.5-fold (*p* < 0.05) with the addition of glucose (Figures 2, 4 and Table 3A). In xylose medium, *C. beijerinckii_*M1GS^DisA^ achieved a maximal butanol titer that is 1.1-fold higher than *C. beijerinckii*_pMTL500E; this change increased to 1.3-fold (*p* < 0.05) with the addition of glucose. Thus, butanol production notably increased by either 50% or 30% in *C. beijerinckii* _M1GS^DisA^ grown in media with either glucose + arabinose or glucose + xylose, respectively, when compared to the control strain; the basis for this solventogenesis difference between the two pentoses remains to be determined by future transcriptomic studies. In contrast, the gains with *C. beijerinckii*_M1GS^CcpA^ were more modest (less than 10% regardless of the combinations tested). In all fermentations, the extent of changes in ABE levels mirrored the corresponding butanol titers (Figures 2, 4 and Table 3). Calculated yield, productivity, and residual sugars in cultures of *C. beijerinckii*_M1GS^CcpA^, _M1GS^DisA^ and _pMTL500E after a 60-h fermentation are consistent with the solvent production profiles (Supplementary Tables 2, 3).

To further characterize the phenotypes of *C. beijerinckii_*M1GS^CcpA^, _M1GS^DisA^, and _pMTL500E, their optical densities (OD_600_) during fermentation on the various carbon substrates mentioned above were measured (Supplementary Figure 2A). Growth for all three strains was comparable in glucose- and arabinose-containing media. *C. beijerinckii_*M1GS^CcpA^ grew well in glucose + arabinose medium, whereas the maximal OD_600_ values of the other two strains (*C. beijerinckii*_M1GS^DisA^ and _pMTL500E) were both ∼1.2-fold lower (*p* < 0.05) than the glucose-containing medium. In glucose + xylose and xylose-alone media, *C. beijerinckii_*M1GS^DisA^ showed the poorest growth, with maximal OD_600_ values up to 1.4-fold lower (*p* < 0.05) than those of *C. beijerinckii*_M1GS^CcpA^ or _pMTL500E grown on different combinations (Supplementary Figure 2A). We could not gain clear insights as to solventogenesis gains based on these growth curves.

### Increased Complementarity in GS^DisA^ Did Not Lead to Solventogenic Gain

Given the enhanced solventogenesis elicited by M1GS^DisA^ even though its GS has only 9-bp complementarity with the DisA mRNA, we then examined whether an M1GS with additional base pairing would be even more effective. To this end, we constructed *C. beijerinckii*_M1GS^DisA16^ that has a full 16-nucleotide complementarity to DisA mRNA. As an additional control, we generated *C. beijerinckii*_ΔM1GS^DisA16^ in which the latter half of M1 RNA was deleted to render it inactive; this control helps determine the antisense effect of the GS portion. We found that DisA mRNA decreased ∼44- and 1.5-fold in *C. beijerinckii*_M1GS^DisA16^ and _ΔM1GS^DisA16^, respectively (Table 2). Despite a comparable knockdown in DisA mRNA level relative to *C. beijerinckii*_M1GS^DisA^ (53-fold), *C. beijerinckii*_M1GS^DisA16^ showed a very different growth and solvent profile relative to *C*. *beijerinckii*_M1GS^DisA^.

The different strains of *C. beijerinckii* studied herein exhibited varying growth profiles with different sugar substrates. For instance, cultures of *C. beijerinckii*_M1GS^DisA16^ reached maximum optical densities at 60 h when grown on glucose and glucose + arabinose, whereas *C. beijerinckii*_M1GS^DisA^, _ΔM1GS^DisA16^, _M1GS^CcpA^, and _pMTL500E reached maximum optical densities at 24 h on glucose and glucose + arabinose, except for *C. beijerinckii*_ΔM1GS^DisA^ that grew to a maximum optical density at 36 h on glucose and arabinose (Supplementary Figures 2A,B). Conversely, when grown on glucose + xylose, _M1GS^DisA16^ attained greater optical density and in considerably less time (36 h), when compared to its growth on glucose or glucose + arabinose (Supplementary Figures 2A,B).

To further assess the effects of DisA knockdown on solvent production, we compared the solvent profiles of *C. beijerinckii*_ΔM1GS^DisA16^ and _M1GS^DisA16^ to those of _M1GS^DisA^, _M1GS^CcpA^, and _pMTL500E in media containing glucose, glucose + arabinose, and glucose + xylose (Figures 2, 4 and Supplementary Figure 3). Like its growth profile (Supplementary Figure 2B), *C. beijerinckii*_M1GS^DisA16^ exhibited a considerable lag in solvent accumulation (Supplementary Figure 3) when grown on glucose or glucose + arabinose but not on glucose + xylose. Notably, *C. beijerinckii*_ΔM1GS^DisA16^ achieved similar maximum solvent concentrations as *C. beijerinckii*_M1GS^DisA16^ and _M1GS^DisA^ when grown on glucose and glucose + xylose (Supplementary Figure 3). However, when all three strains were grown on glucose + arabinose, *C. beijerinckii*_M1GS^DisA^ produced significantly more butanol and ABE than *C. beijerinckii*_ΔM1GS^DisA16^ and _M1GS^DisA16^.

## Discussion

**FIGURE 5:**
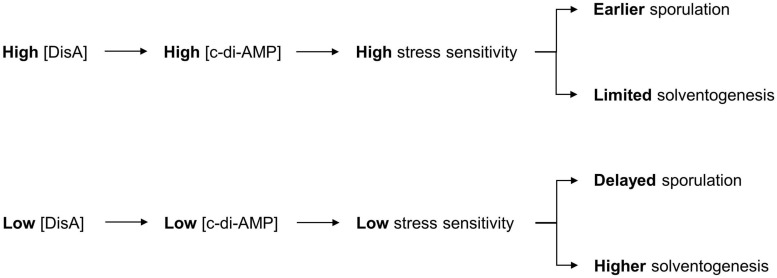
Model for the role of c-di-AMP in ABE production. The depiction includes the normal sequence of events during c-di-AMP production in *C. beijerinckii* and the role of c-di-AMP in the initiation of sporulation, which impacts the duration of ABE biosynthesis. Decrease in c-di-AMP production following DisA knockdown in *C. beijerinckii*_M1GS^DisA^ would delay activation of Spo0A, and hence, the onset of sporulation. This delay would therefore extend the solventogenic phase, thereby allowing increased accumulation of ABE pre-sporulation.

Although we fulfilled our goal of improving ABE production in *C. beijerinckii* grown on mixed sugars, it was accomplished more through fortuitous knockdown of DisA than of CcpA, which was our intended target originally for dampening CCR. While a modest ABE increase was observed with CcpA downregulation, our results provide insights into the M1GS-based knockdown approach in *C. beijerinckii* and the hitherto unknown role of DisA (via c-di-AMP) in solventogenesis (Figure 5).

The M1GS-based mRNA degradation approach has been used successfully to cleave mRNAs in bacteria, mammalian cells, and mice (Liu, 2010). Nevertheless, our finding of the 53-fold decrease in the DisA mRNA level in *C. beijerinckii* elicited by M1GS^DisA^ is notable for a few reasons. First, we show the utility of this method in an anaerobic, Gram-positive bacterium and demonstrate its merit as a tool for studying the physiology and metabolic engineering of *Clostridium* species. Second, our copy number determination of the DisA mRNA and M1GS^DisA^ confirms the unproven expectation of multiple turnover by this ribozyme *in vivo* (Table 2). Third, the M1GS method typically aims for 13-15 base pairs between the GS and the target mRNA to achieve optimal binding and cleavage, with the caveat that long stems favorable for GS-mRNA complex formation might inhibit turnover by preventing product release. That M1GS^DisA^ can form only 9 base pairs with the DisA mRNA (Figure 1C) may account for the efficient turnover that we observed; it is possible that intracellular factors (e.g., chaperones) contribute favorably either to the annealing and thermodynamic stability of the GS-mRNA complex or to product dissociation. While such short GS-mRNA duplexes raise the specter of off-target effects, this concern is partly alleviated by accessibility considerations. In fact, the degree of knockdown observed for DisA mRNA suggests that the target sequence on this mRNA is accessible. This expectation is consistent with our observation that extending the GS-DisA mRNA complementarity from 9- (Figure 1C) to 16-base pairs (Figure 1D) resulted in a similar extent of DisA mRNA knockdown (∼44-fold), relative to the plasmid control strain (*C. beijerinckii*_pMTL500E). Fourth, these DisA-targeted GSs bind to the 334-349 region of DisA mRNA (numbering with reference to the start codon; Figures 1C,D), whereas GSs are typically designed to target nucleotides immediately downstream of the start codon because they are often single-stranded to ensure high translatability. The efficient knockdown with these DisA-targeted GSs suggests that local secondary structure elsewhere in the mRNA, which is hard to predict under cellular conditions, might offer more accessible targets (Cobaleda and Sánchez-García, 2000; Lundblad et al., 2008). New methods that map RNA secondary structure *in vivo* will be valuable in this regard (Spitale et al., 2013; Watters et al., 2016; Zhao et al., 2019). Last, *in vitro* studies suggest that M1GS prefers to cleave between a pyrimidine and a guanosine (Figure 1B; Li and Altman, 1996), but this cleavage site requirement is not absolute. Indeed, based on the predicted targeting site in the DisA mRNA (Figures 1C,D), it appears that this is not a strict requirement *in vivo*.

It is surprising that while we set out to knock down CcpA in *C. beijerinckii* to abolish or at least dampen CCR, we achieved only a 3-fold decrease in the CcpA mRNA level with M1GS^CcpA^ when M1GS^DisA^ could reduce the DisA mRNA level by 53-fold. Nevertheless, the 3-fold decrease in CcpA mRNA levels was accompanied by a 10-20% increase in ABE production compared to the control when grown on arabinose, glucose + arabinose, or glucose + xylose media (Figures 2, 4 and Table 3). The fact that the increase was associated with pentose-containing media suggests that M1GS^CcpA^ was able to dampen CCR. That M1GS^CcpA^ was not more effective could be explained by gene duplication in *C. beijerinckii* (Hall et al., 2009; Shi et al., 2010). While our customized M1GS^CcpA^ was designed to target *Cbei_0047*, which is annotated to encode a CcpA/alanine racemase, the *C. beijerinckii* genome has seven other genes annotated to encode putative alanine racemases that have sequence similarity to *Cbei_0047* as well as to the *C. acetobutylicum* CcpA (Supplementary Table 4). Given that all these seven proteins harbor DNA-binding helix-turn-helix and sugar-binding domains like those found in *Cbei_0047*, a 3-fold downregulation of the CcpA mRNA encoded by *Cbei_0047* may not be sufficient to eliminate CCR.

Decreased DisA mRNA and c-di-AMP levels, increased sensitivity to DNA-damaging antibiotics, and delayed sporulation in *C. beijerinckii*_M1GS^DisA^ are consistent with the 53-fold knockdown of DisA mRNA. However, the 7-fold decrease in cellular c-di-AMP level in *C. beijerinckii*_M1GS^DisA^ was lower than expected but easily rationalized. While most organisms have only one diadenylate cyclase (DAC; a c-di-AMP–producing enzyme), *Clostridium* and *Bacillus* species possess two to three DACs, all subject to spatio-temporal regulation (Corrigan and Gründling, 2013; Mehne et al., 2013). In fact, the presence of c-di-AMP in a DisA-null mutant of *B. subtilis* during vegetative growth confirmed the involvement of other DACs in c-di-AMP production (Oppenheimer-Shaanan et al., 2011). To determine if there are additional DACs in the *C. beijerinckii* genome, we searched for proteins annotated with a DAC domain (DUF147; Mehne et al., 2013). In addition to *Cbei_0127* (DisA), we found only *Cbei_0200*, a conserved hypothetical protein, to have this domain. *Cbei_0200* was also the only *C. beijerinckii* protein uncovered in a BLASTp search when the sequences of *Cbei_0127* (DisA) and the three *B. subtilis* DACs (CdaA, CdaS, and DisA) were each used as a query. While the protein product of *Cbei_0200* might be a DAC homolog that contributes to intracellular c-di-AMP levels, it is unlikely that *Cbei_0200* encodes a DisA homolog as it shares only 22% similarity with *Cbei_0127*. Importantly, the poor complementarity between *Cbei_0200* and GS^DisA^ would allow *Cbei_0200* to escape downregulation by M1GS^DisA^ and to continue contributing to the cellular c-di-AMP level in *C. beijerinckii_*M1GS^DisA^. As in *B. subtilis*, where DisA is expressed predominantly at high cell density and at the onset of sporulation (Bejerano-Sagie et al., 2006; Mehne et al., 2013), decreases in the c-di-AMP level in *C. beijerinckii*_M1GS^DisA^ relative to the control strains were pronounced at high cell density (7-fold at 24 h of growth) and at the peak of sporulation (5-fold at 60 h; Figure 3A and Supplementary Figure 4).

In some media, *C. beijerinckii_*M1GS^DisA^ grew slower but produced more ABE than other strains (Figures 2, 4 and Supplementary Figures 2, 3). c-di-AMP is essential for growth in rich media but is dispensable in minimal media (Whiteley et al., 2015). Decreased c-di-AMP level is the likely cause for the poorer growth in the first 24 h when nutrients in the media is high. We can also rationalize our observation that a more efficient channeling of carbon to ABE biosynthesis over biomass accumulation exists in this strain, a desirable trait for large-scale fermentation. As c-di-AMP can directly inactivate pyruvate carboxylase (Sureka et al., 2014), decreasing the level of this secondary messenger could support the TCA cycle and thereby solventogenesis in *C. beijerinckii_*M1GS^DisA^. In addition, high c-di-AMP level leads to increased stress sensitivity, especially to high salt concentrations (and, perhaps, other stressors including ABE products); the low c-di-AMP level in *C. beijerinckii_*M1GS^DisA^ likely mitigated such stress sensitivity. Coupled to the delayed onset of sporulation mediated by DisA knockdown (Figure 3C), solvent production in *C. beijerinckii_*M1GS^DisA^ can continue unabated longer than other strains.

While *C. beijerinckii*_M1GS^DisA^ and _M1GS^DisA16^ showed a similar degree of DisA mRNA fold change (∼44-fold decrease; Table 2), their growth and solvent profiles (Figures 2, 4) are seemingly disparate but share a pattern. First, their slow growth, though worse in the latter strain, occurred only at the early stages of culturing. Second, while higher solvent production is evident in *C. beijerinckii_*M1GS^DisA^ after 12 h of growth, *C. beijerinckii_*M1GS^DisA16^ displayed an initial lag but the ABE production increased thereafter to at least a level that matched that of other strains. Both observations could be explained by decreased c-di-AMP levels as discussed above: poor growth in rich media and a rapid increase in ABE productivity in later stages. The reason for differences in phenotypes between *C. beijerinckii_*M1GS^DisA^ and *C. beijerinckii_*M1GS^DisA16^ is unclear but may involve stronger off-target effects by GS^DisA16^. Support for this possibility comes from the observation that of the five mRNAs encoding transporters examined by RT-qPCR as possible off-targets of M1GS^DisA^ (Supplementary Table 1), three (including ABC transporter component of monosaccharide transport system and ATP-binding cassette domain of the histidine-glutamine transporters) showed several-fold downregulation. These findings suggest that for organisms with AT-rich genomes such as *C. beijerinckii*, promiscuity with the M1GS approach is inevitable. Even a naturally occurring small RNA in solventogenic clostridia was found to be a pleiotropic regulator due to its ability to target multiple mRNAs simultaneously (Yang et al., 2020). Thus, the specificity issues that we found unpredictable might be attributable to the compositional make-up of the transcriptome and repertoire of cellular factors in *C. beijerinckii*.

Asporogenic strains of solventogenic clostridia have long been considered ideal for continuous fermentation and longer-lasting batch fermentation with improved sugar utilization and ABE production. However, obtaining such a strain has thus far proven elusive. Our observation of enhanced ABE production by knocking down a gene not directly involved in the onset of sporulation (Figure 5) merits further investigation and highlights the importance of understanding the intricate coupling between sporulation and solventogenesis. While DisA knockdown exhibited improved ABE production, it also increased sensitivity to DNA damage (Figure 3B). Future studies to decrease intracellular c-di-AMP levels either by downregulating other DACs (not involved in genome scanning and DNA repair) or by upregulating the c-di-AMP phosphodiesterases may be a viable and appealing alternative.

Overall, our results confirm that DisA is a significant player in the regulation of solvent production in *C. beijerinckii*. It is plausible that a similar phenomenon exists in other solventogenic *Clostridium* species. While our findings demonstrate the utility of knockdown over knockout, the confounding issue of off-target effects need to be addressed before deployment of the ribozyme approach in solventogenic *Clostridium* species. Such concerns may be partly offset as long as the defined payoffs are accomplished as illustrated here for solventogenesis. Regardless, the M1GS ribozyme approach could be harnessed to uncover new determinants of phenotypes such as growth, solventogenesis, and sporulation, and inspire targeted genetic manipulations to boost ABE production. Indeed, this work shows that MIGS technology has the potential of being a metabolic engineering tool of solventogenic *Clostridium* species, and perhaps, other microorganisms. The recent demonstration of the utility of the endogenous type IB CRISPR-Cas machinery as a powerful tool for engineering solventogenic *Clostridium* species (Payne et al., 2016; Zhang et al., 2018; Atmadjaja et al., 2019) further highlights the potential for using the RNase P-based approach as a gene-knockdown complement to a knockout method.

## Data Availability Statement

The original contributions presented in the study are included in the article/Supplementary Material, further inquiries can be directed to the corresponding author/s.

## Author Contributions

TE and VG conceived and designed the study. VU, LL, and CO performed the experiments. VU analyzed the data. VU and LL wrote the first draft of the manuscript. All authors contributed to the writing of the final draft and approved the submitted version.

## Conflict of Interest

The authors declare that the research was conducted in the absence of any commercial or financial relationships that could be construed as a potential conflict of interest.

## References

[B1] Altman S. (2007). A view of RNase P. *Mol. Biosyst.* 3 604–607. 10.1039/b707850c 17700860

[B2] Atmadjaja A. N. Holby V. Harding A. J. Krabben P. Smith H. K. Jenkinson E. R. (2019). CRISPR-Cas, a highly effective tool for genome editing in *Clostridium saccharoperbutylacetonicum* N1-4(HMT). *FEMS Microbiol. Lett.* 366:fnz059. 10.1093/femsle/fnz059 30874768 PMC6491355

[B3] Bai Y. Gong H. Li H. Vu G. P. Lu S. Liu F. (2011). Oral delivery of RNase P ribozymes by *Salmonella* inhibits viral infection in mice. *Proc. Natl. Acad. Sci. U.S.A.* 108 3222–3227. 10.1073/pnas.1014975108 21300908 PMC3044408

[B4] Bai Y. Rider P. J. Liu F. (2010). Catalytic M1GS RNA as an antiviral agent in animals. *Methods Mol. Biol.* 629 339–353. 20387160 10.1007/978-1-60761-657-3_22

[B5] Bejerano-Sagie M. Oppenheimer-Shaanan Y. Berlatzky I. Rouvinski A. Meyerovich M. Ben-Yehuda S. (2006). A checkpoint protein that scans the chromosome for damage at the start of sporulation in *Bacillus subtilis*. *Cell* 125 679–690. 10.1016/j.cell.2006.03.039 16713562

[B6] Cobaleda C. Sánchez-García I. (2000). In vivo inhibition by a site-specific catalytic RNA subunit of RNase P designed against the BCR-ABL oncogenic products: a novel approach for cancer treatment. *Blood* 95 731–737. 10.1182/blood.v95.3.731.003k28_731_737 10648380

[B7] Corrigan R. M. Gründling A. (2013). Cyclic di-AMP: another second messenger enters the fray. *Nat. Rev. Microbiol.* 11 513–524. 10.1038/nrmicro3069 23812326

[B8] Devonshire A. S. Sanders R. Whale A. S. Nixon G. J. Cowen S. Ellison S. L. R. (2016). An international comparability study on quantification of mRNA gene expression ratios: CCQM-P103.1. *Biomol. Detect. Quantif.* 8 12–28. 10.1016/j.bdq.2016.05.003 PMC4906133 27335807

[B9] Ezeji T. C. Groberg M. Qureshi N. Blaschek H. P. (2003). Continuous production of butanol from starch-based packing peanuts. *Appl. Biochem. Biotechnol.* 108 375–382. 10.1385/abab:106:1-3:375 12721460

[B10] Ezeji T. C. Milne C. Price N. D. Blaschek H. P. (2010). Achievements and perspectives to overcome the poor solvent resistance in acetone and butanol-producing microorganisms. *Appl. Microbiol. Biotechnol.* 85 1697–1712. 10.1007/s00253-009-2390-0 20033401

[B11] Ezeji T. C. Qureshi N. Blaschek H. P. (2007). Butanol production from agricultural residues: impact of degradation products on *Clostridium beijerinckii* growth and butanol fermentation. *Biotechnol. Bioeng.* 97 1460–1469. 10.1002/bit.21373 17274071

[B12] Forster A. C. Altman S. (1990). External guide sequence for an RNA enzyme. *Science* 249 783–786. 10.1126/science.1697102 1697102

[B13] Gopalan V. Vioque A. Altman S. (2002). RNase P: variations and uses. *J. Biol. Chem.* 277 6759–6762. 10.1074/jbc.r100067200 11741968

[B14] Grupe H. Gottschalk G. (1992). Physiological events in *Clostridium acetobutylicum* during the shift from acidogenesis to solventogenesis in continuous culture and presentation of a model for shift induction. *Appl. Environ. Microbiol.* 58 3896–3902. 10.1128/aem.58.12.3896-3902.1992 16348821 PMC183201

[B15] Guerrier-Takada C. Altman S. (2000). Inactivation of gene expression using ribonuclease P and external guide sequences. *Methods Enzymol.* 313 442–456. 10.1016/s0076-6879(00)13028-9 10595372

[B16] Hall B. G. Pikis A. Thompson J. (2009). Evolution and biochemistry of family 4 glycosidases: implications for assigning enzyme function in sequence annotations. *Mol. Biol. Evol.* 26 2487–2497. 10.1093/molbev/msp162 19625389 PMC2767093

[B17] Ho N. W. Chen Z. Brainard A. P. (1998). Genetically engineered Saccharomyces yeast capable of effective cofermentation of glucose and xylose. *Appl. Environ. Microbiol.* 64 1852–1859. 10.1128/aem.64.5.1852-1859.1998 9572962 PMC106241

[B18] Jones D. T. Woods D. R. (1986). Acetone-butanol fermentation revisited. *Microbiol. Rev.* 50 484–524. 10.1128/mr.50.4.484-524.1986 3540574 PMC373084

[B19] Khvorova A. Lescoute A. Westhof E. Jayasena S. D. (2003). Sequence elements outside the hammerhead ribozyme catalytic core enable intracellular activity. *Nat. Struct. Biol.* 10 708–712. 10.1038/nsb959 12881719

[B20] Kim A. Y. Blaschek H. P. (1993). Construction and characterization of a phage-plasmid hybrid (phagemid), pCAK1, containing the replicative form of virus-like particle CAKi isolated from *Clostridium acetobutylicum* NCIB 64444. *J. Bacteriol.* 175 3838–3842. 10.1128/jb.175.12.3838-3843.1993 8509336 PMC204800

[B21] Lai L. B. Vioque A. Kirsebom L. A. Gopalan V. (2010). Unexpected diversity of RNase P, an ancient tRNA processing enzyme: challenges and prospects. *FEBS Lett.* 584 287–296. 10.1016/j.febslet.2009.11.048 19931535 PMC2799185

[B22] Leboeuf C. Leblanc L. Auffray Y. Hartke A. (2000). Characterization of the CcpA gene of Enterococcus faecalis: identification of starvation-inducible proteins regulated by CcpA. *J. Bacteriol.* 182 5799–5806. 10.1128/jb.182.20.5799-5806.2000 11004180 PMC94703

[B23] Li Y. Altman S. (1996). Cleavage by RNase P of gene N mRNA reduces bacteriophage lambda burst size. *Nucleic Acids Res.* 24 835–842. 10.1093/nar/24.5.835 8600449 PMC145720

[B24] Liu F. (2010). “Ribonuclease P as a tool,” in *Ribonuclease P*, eds Liu F. Altman S. (New York: Springer), 153–172.

[B25] Liu F. Altman S. (1995). Inhibition of viral gene expression by the catalytic RNA subunit of RNase P from *Escherichia coli*. *Genes Dev.* 9 471–480. 10.1101/gad.9.4.471 7533740

[B26] Ludwig H. Meinken C. Martin A. Stulke J. (2002). Insufficient expression of the ilv-leu operon encoding enzymes and branched-chain amino acid biosynthesis limits growth of *Bacillus subtilis* CcpA mutant. *J. Bacteriol.* 184 5174–5178. 10.1128/jb.184.18.5174-5178.2002 12193635 PMC135319

[B27] Lundblad E. W. Xiao G. Ko J. H. Altman S. (2008). Rapid selection of accessible and cleavable sites in RNA by *Escherichia coli* RNase P and random external guide sequences. *Proc. Natl. Acad. Sci. U.S.A.* 105 2354–2357. 10.1073/pnas.0711977105 18263737 PMC2268140

[B28] Mazzeo M. F. Cacace G. Peluso A. Zotta T. Muscariello L. Vastano V. (2012). Effect of inactivation of CcpA and aerobic growth in *Lactobacillus plantarum*: A proteomic perspective. *J. Proteomics* 75 4050–4061. 10.1016/j.jprot.2012.05.019 22634038

[B29] Mehne F. M. P. Gunka K. Eilers H. Herzberg C. Kaever V. Stülke J. (2013). Cyclic di-AMP homeostasis in *Bacillus subtilis*: both lack and high level accumulation of the nucleotide are detrimental for cell growth. *J. Biol. Chem.* 288 2004–2017. 10.1074/jbc.m112.395491 23192352 PMC3548507

[B30] Mitchell W. J. (1998). Physiology of carbohydrate to solvent conversion by clostridia. *Adv. Microb. Physiol.* 39 31–130. 10.1016/s0065-2911(08)60015-6 9328646

[B31] Oppenheimer-Shaanan Y. Wexeselblatt E. Katzhendler J. Yavin E. Ben-Yehuda S. (2011). c-di-AMP reports DNA integrity during sporulation in *Bacillus subtilis*. *EMBO Rep.* 12 594–601. 10.1038/embor.2011.77 21566650 PMC3128283

[B32] Ounine K. Petitdemange H. Raval G. Gay G. (1985). Regulation and butanol inhibition of D-xylose and D-glucose uptake in *Clostridium acetobutylicum*. *Appl. Environ. Microbiol.* 49 874–878. 10.1128/aem.49.4.874-878.1985 4004220 PMC238462

[B33] Payne M. E. Bruder M. R. Moo-Young M. Chung D. A. Chou C. P. (2016). Harnessing heterologous and endogenous CRISPR-Cas machineries for efficient markerless genome editing in *Clostridium*. *Sci. Rep.* 6:25666. 27157668 10.1038/srep25666 PMC4860712

[B34] Przybilski R. Gräf S. Lescoute A. Nellen W. Westhof E. Steger G. (2005). Functional hammerhead ribozymes naturally encoded in the genome of *Arabidopsis thaliana*. *Plant Cell* 17 1877–1885. 10.1105/tpc.105.032730 15937227 PMC1167538

[B35] Ren C. Gu Y. Hu S. Wu Y. Wang P. Yang Y. (2010). Identification and inactivation of pleiotropic regulator CcpA to eliminate glucose repression of xylose utilization in *Clostridium acetobutylicum*. *Metab. Eng.* 12 446–454. 10.1016/j.ymben.2010.05.002 20478391

[B36] Ren C. Gu Y. Wu Y. Zhang W. Yang C. Yang S. (2012). Pleiotropic functions of catabolite control protein CcpA in butanol-producing *Clostridium acetobutylicum*. *BMC Genomics* 13:349. 10.1186/1471-2164-13-349 22846451 PMC3507653

[B37] Scott C. W. Engelke D. R. (2006). Ribonuclease P: the evolution of an ancient RNA enzyme. *Crit. Rev. Biochem. Mol. Biol.* 41 77–102. 10.1080/10409230600602634 16595295 PMC2803672

[B38] Shi Y. Li Y. X. Li Y. Y. (2010). Large number of phosphotransferase gens in the *Clostridium beijerinckii* NCIMB 8052 genome and study on their evolution. *BMC Bioinformatics* 11(Suppl. 11):S9. 10.1186/1471-2105-11-S11-S9 PMC3024867 21172059

[B39] Siemerink A. J. Kuit W. López-Contreras A. M. Eggink G. van der Oost J. Kengen S. W. M. (2011). D-2,3-butanediol production due to heterologous expression of an acetoin reductase in *Clostridium acetobutylicum*. *Appl. Environ. Microbiol.* 77 2582–2588. 10.1128/aem.01616-10 21335380 PMC3126369

[B40] Singh K. D. Schmalisch M. H. Stülke J. Görke B. (2008). Carbon catabolite repression in *Bacillus subtilis*: quantitative analysis of repression exerted by different carbon sources. *J. Bacteriol.* 190 7275–7284. 10.1128/jb.00848-08 18757537 PMC2580719

[B41] Spitale R. C. Crisalli P. Flynn R. A. Torre E. A. Kool E. T. Chang H. Y. (2013). RNA SHAPE analysis in living cells. *Nat. Chem. Biol.* 9 18–20. 10.1038/nchembio.1131 23178934 PMC3706714

[B42] Stülke J. Krüger L. (2020). Cyclic di-AMP signaling in bacteria. *Annu. Rev. Microbiol.* 74 159–179. 10.1146/annurev-micro-020518-115943 32603625

[B43] Sureka K. Choi P. H. Precit M. Delince M. Pensinger D. A. Huynh T. N. (2014). The cyclic dinucleotide c-di-AMP is an allosteric regulator of metabolic enzyme function. *Cell* 158 1389–1401. 10.1016/j.cell.2014.07.046 25215494 PMC4166403

[B44] Tsai H. Y. Lai L. B. Gopalan V. (2002). A modified Bluescript vector for facile cloning and transcription of RNA. *Anal. Biochem.* 303 214–217. 10.1006/abio.2001.5567 11950224

[B45] Uhlenbeck O. C. (1987). A small catalytic oligoribonucleotide. *Nature* 328 596–600. 10.1038/328596a0 2441261

[B46] Ujor V. Agu C. V. Gopalan V. Ezeji T. C. (2014). Glycerol supplementation enhances furfural detoxification by *Clostridium beijerinckii* during butanol fermentation. *Appl. Microbiol. Biotechnol.* 98 6511–6521. 10.1007/s00253-014-5802-8 24839212

[B47] Vioque A. Arnez J. Altman S. (1988). Protein-RNA interactions in the RNase P holoenzyme from *Escherichia coli*. *J. Mol. Biol.* 202 835–848. 10.1016/0022-2836(88)90562-1 2459398

[B48] Watters K. E. Abott T. R. Lucks J. B. (2016). Simultaneous characterization of cellular RNA structure and function with in-cell SHAPE-Seq. *Nucleic Acids Res.* 44:e12. 10.1093/nar/gkv879 26350218 PMC4737173

[B49] Whiteley A. T. Pollock A. J. Portnoy D. A. (2015). The PAMP c-di-AMP is essential for Listeria monocytogenes growth in rich but not minimal media due to a toxic increase in (p)ppGpp. *Cell Host Microbe* 17 788–798. 10.1016/j.chom.2015.05.006 26028365 PMC4469362

[B50] Yang Y. Zhang H. Lang N. Zhang L. Chai C. He H. (2020). The small RNA sr8384 is a crucial regulator of cell growth in solventogenic clostridia. *Appl. Envi. Microbiol.* 86:e00665-20. 10.1128/AEM.00665-20 PMC7301862 32358006

[B51] Zhang J. Zong W. Hong W. Zhang Z.-T. Wang Y. (2018). Exploiting endogenous CRISPR-Cas system for multiplex genome editing in *Clostridium tyrobutyricum* and engineer the strain for high-level butanol production. *Metab. Eng.* 47 49–59. 10.1016/j.ymben.2018.03.007 29530750

[B52] Zhang Y. Ezeji T. C. (2013). Transcriptional analysis of *Clostridium beijerinckii* NCIMB 8052 to elucidate the role of furfural stress during acetone butanol fermentation. *Biotechnol. Biofuels* 6 66–82. 10.1186/1754-6834-6-66 23642190 PMC3681630

[B53] Zhao J. Qian X. Yeung P. Y. Zhang Q. F. Kwok C. K. (2019). Mapping in vivo RNA structures and interactions. *Trends Biochem. Sci.* 44 555–556. 10.1016/j.tibs.2019.01.012 30853244

[B54] Zuker M. (2003). Mfold web server for nucleic acid folding and hybridization prediction. *Nucleic Acids Res.* 31 3406–3415. 10.1093/nar/gkg595 12824337 PMC169194

[B55] Zverlov V. V. Berezina O. Velikodvorskaya G. A. Schwarz W. H. (2006). Bacterial acetone and butanol production by industrial fermentation in the Soviet Union: use of hydrolyzed agricultural waste for biorefinery. *Appl. Microbiol. Biotechnol.* 71 587–597. 10.1007/s00253-006-0445-z 16685494

