# Supplementary material for: Ribozyme-Mediated Downregulation Uncovers DNA Integrity Scanning Protein A (DisA) as a Solventogenesis Determinant in Clostridium beijerinckii
Source: Front Bioeng Biotechnol. 2021 Jun 8;9:669462. doi: 10.3389/fbioe.2021.669462 (PMC8217750; doi:10.3389/fbioe.2021.669462)
Supplement: Supplementary file 1 [file Data_Sheet_1.pdf]

Supplementary material: Ujor *et al*

Ribozyme-mediated downregulation uncovers DNA integrity scanning protein A (DisA) as a solventogenesis determinant in *Clostridium beijerinckii*

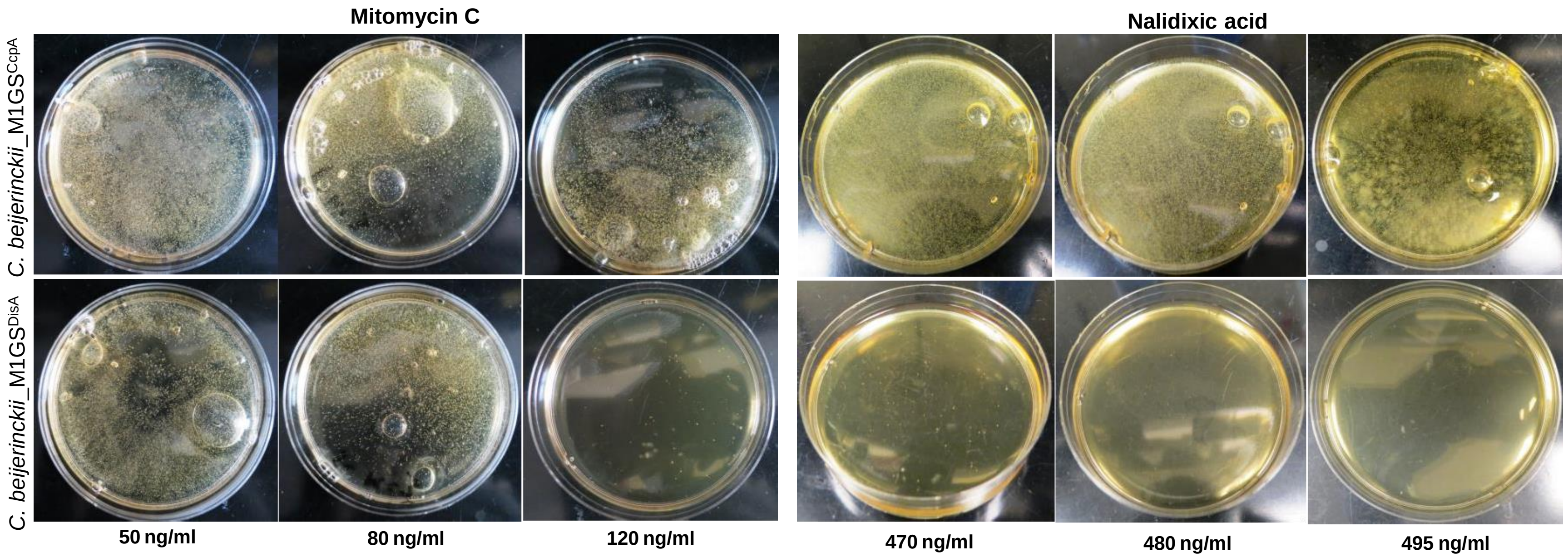

Supplementary Figure 1. Dose-dependent effect of mitomycin C and nalidixic acid on *C. beijerinckii*_M1GS^{CcpA} and _M1GS^{DisA}. Cells were plated on tryptone-glucose-yeast extract (TGY) agar [0.45% (w/v) agar]. Generation of carbon dioxide and hydrogen during cellular growth andmetabolism causes bubbles, made evident by the semi-solid, jelly nature of the medium.

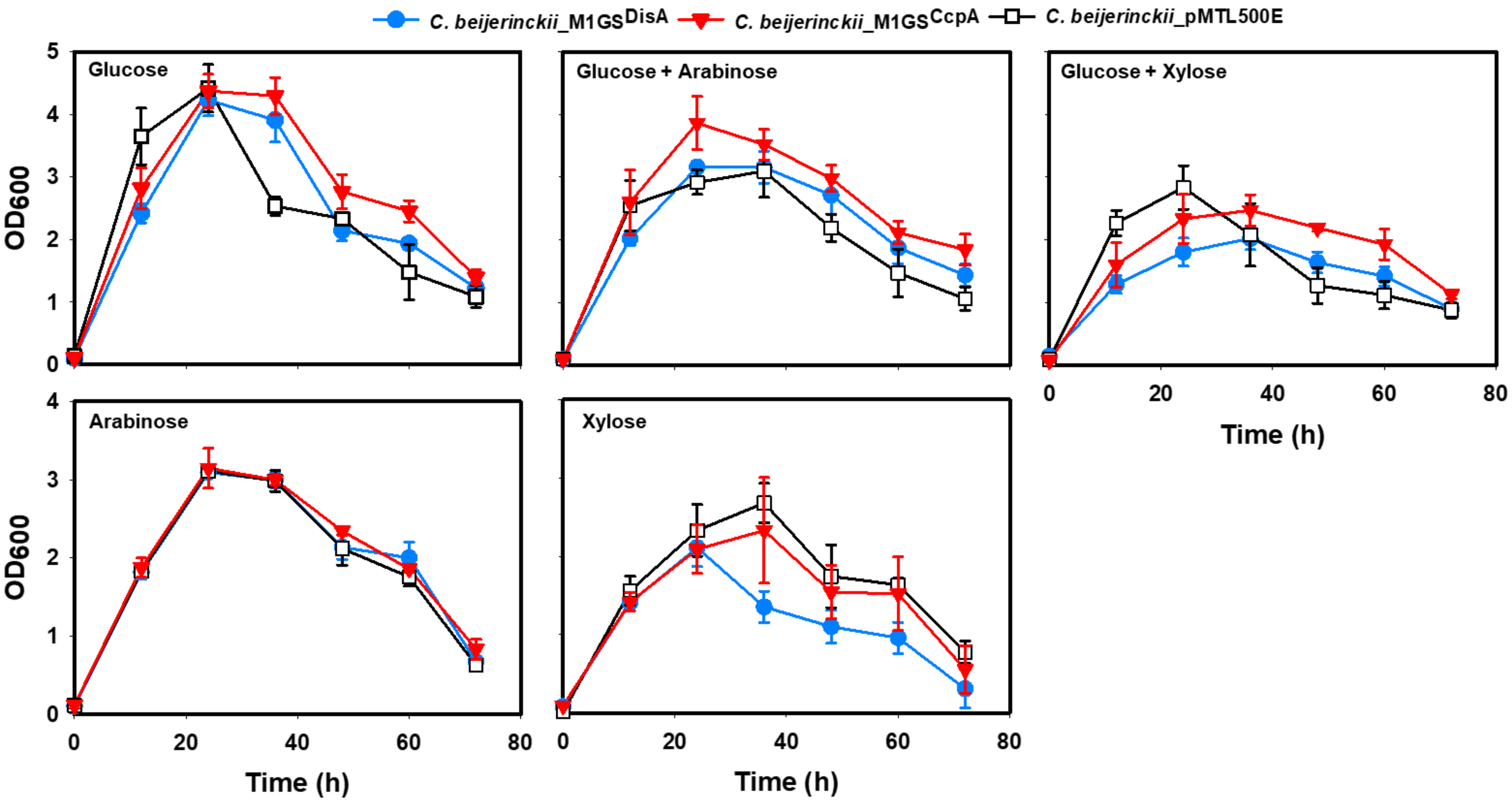

Supplementary Figure 2A. The growth profiles of *C. beijerinckii*_M1GS^{DisA}, _M1GS^{CcpA}, and pMTL500E on glucose, glucose + arabinose, glucose + xylose, arabinose, and xylose during a 72-hour fermentation. Error bars represent standard deviation of the mean (n = 3).

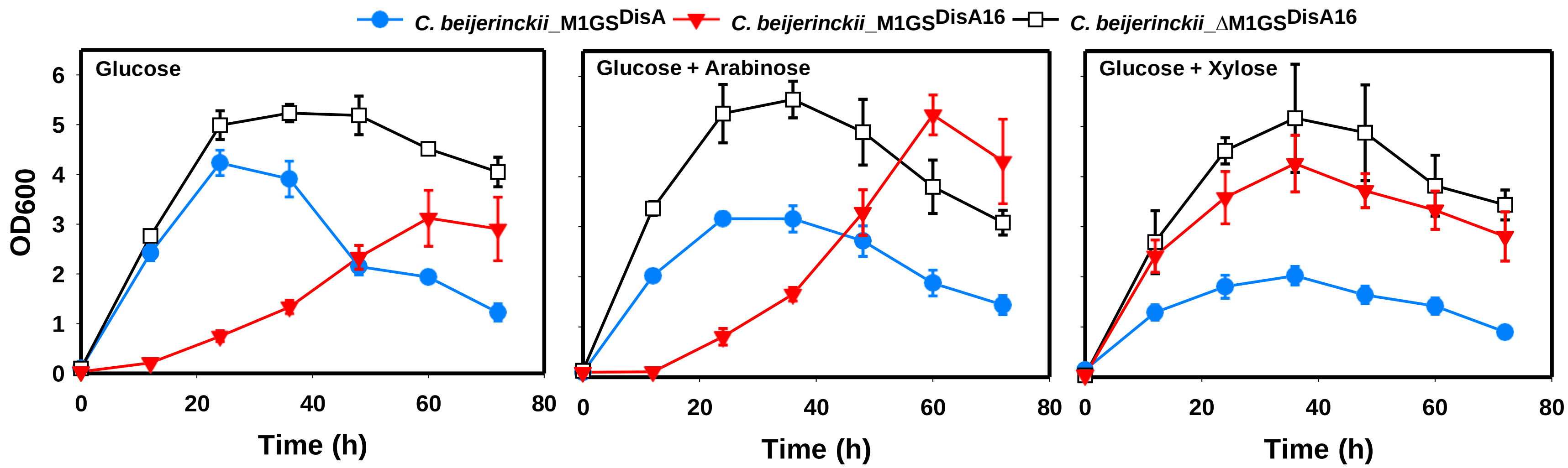

Supplementary Figure 2B. The growth profiles of *C. beijerinckii*_M1GS^{DisA}, _MIGS^{DisA16}, and ΔM1GS^{DisA16} on glucose, glucose + arabinose, and glucose + xylose during a 72-hour fermentation. Error bars represent standard deviation of the mean (n = 3).

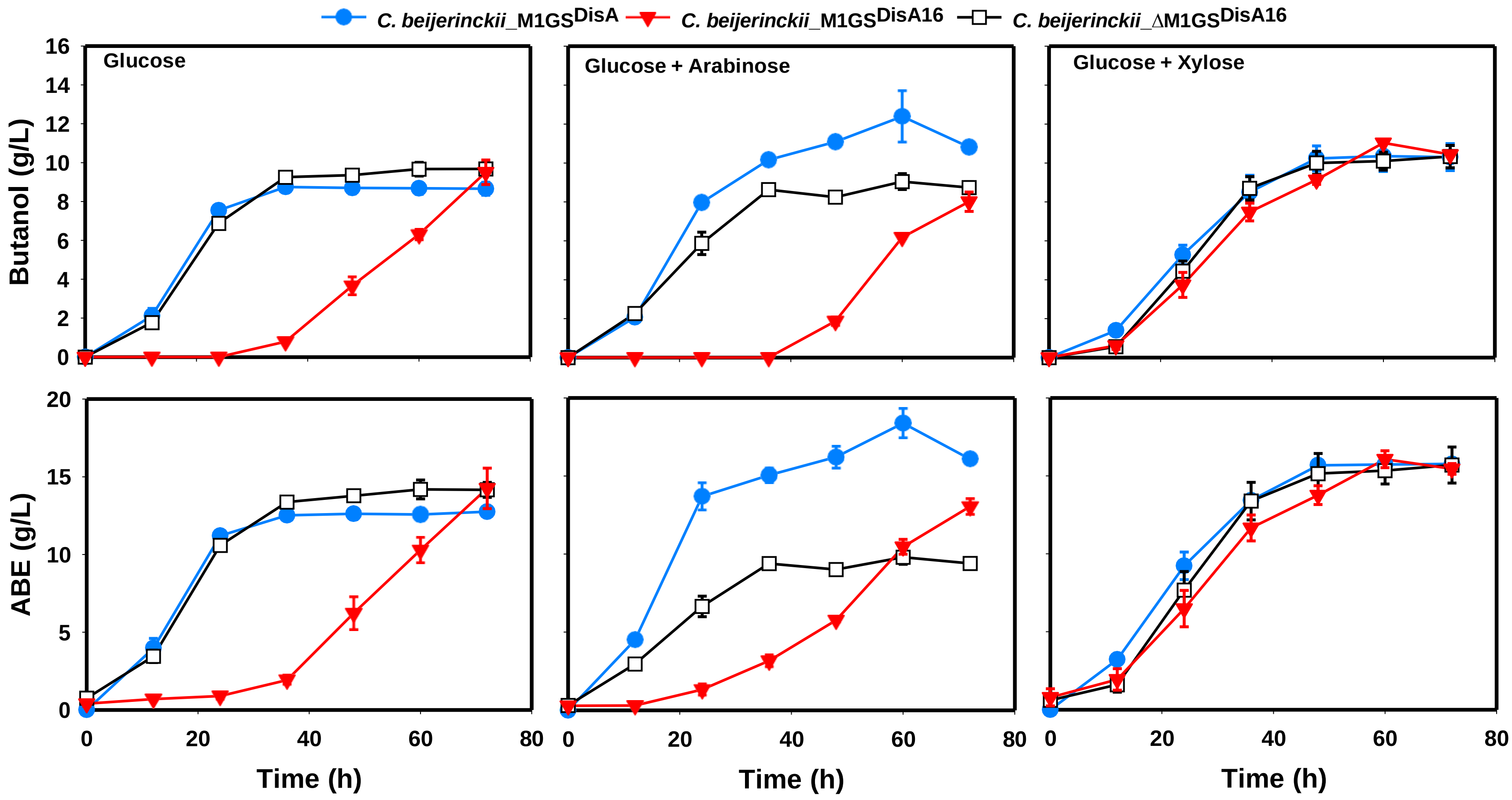

Supplementary Figure 3. Butanol and ABE profiles of *C. beijerinckii*_M1GS^{DisA}, _M1GS^{DisA16} and _ΔM1GS^{DisA} grown on glucose, glucose +arabinose, and glucose + xylose. Error bars represent standard deviation of the mean (n = 3).

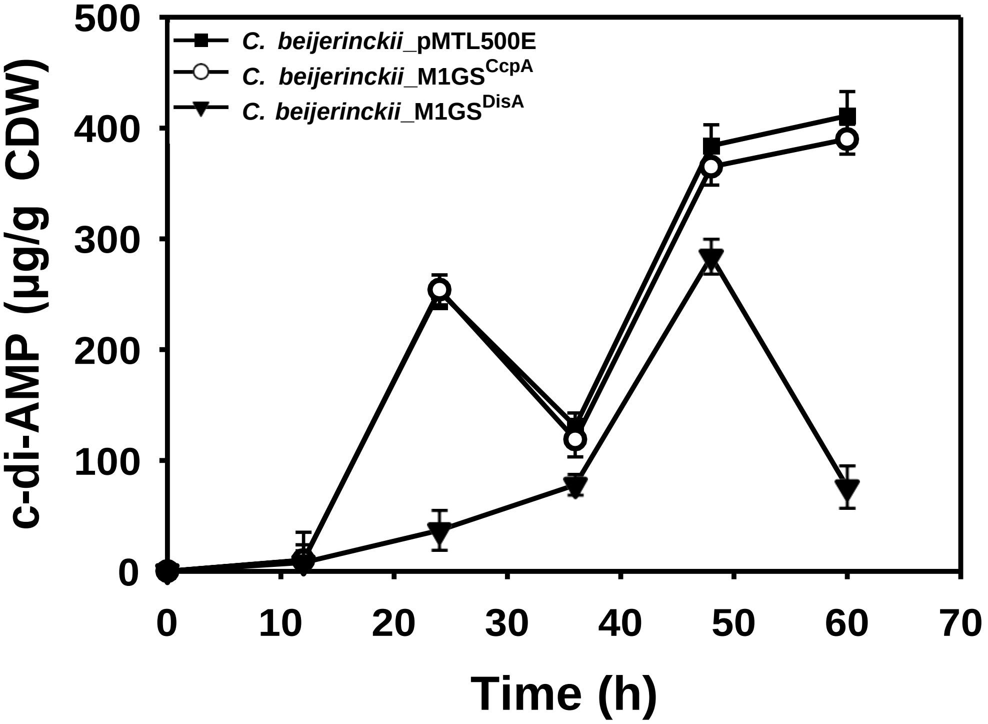

Supplementary Figure 4. The levels of c-di-AMP at 12-h intervals in cultures of *C. beijerinckii*_pMTL500E, _M1GS^{CcpA}, and _M1GS^{DisA} grown in glucose + arabinose medium. Error bars denote standarddeviations (n = 3). CDW, cell dry weight.

Supplementary Table 1. Changes in levels of mRNAs with sequences complementary to $GS^{CcpA\text{-}Sc}$ (GS^{DisA})

mRNA	Putative function	Fold change
Cbei_1775	Phosphate transporter	NSC
Cbei_0380	PTS system lactose/cellobiose-specific transporter subunit IIA	NSC
Cbei_2512	ATP-binding cassette domain of the histidine and glutamine transporters	-5.0 ± 1.2
Cbei_4461	ABC transporter component of monosaccharide transport system/sugar transport and metabolism	-3.5 ± 0.6
Cbei_0182	ATP-binding cassette component of cobalt transport system	-4.2 ± 1.3
Cbei_0127	DNA integrity scanning protein A - DisA (non-specific DNA-binding)	-44.0 ± 3.8

NSC, no significant change

Supplementary Table 2. Butanol and ABE yields and productivities of the three *C. beijerinckii* strains grown on various sugars for 60 h.

Substrate	Solvent	***C. beijerinckii*_pMTL500E**		***C. beijerinckii*_M1GS^{CcpA}**		***C. beijerinckii*_M1GS^{DisA}**	
		Yield (g/g)	Productivity (g/L.h)	Yield (g/g)	Productivity (g/L.h)	Yield (g/g)	Productivity (g/L.h)
Glucose	Butanol	0.24 ± 0.001^{a}	0.21 ± 0.006^{a}	0.21 ± 0.005^{b}	0.20 ± 0.001^{a}	0.25 ± 0.003^{a}	0.24 ± 0.002^{b}
	ABE	0.37 ± 0.002^{a}	0.32 ± 0.004^{a}	0.32 ± 0.007^{b}	0.30 ± 0.002^{a}	0.35 ± 0.004^{a}	0.35 ± 0.003^{b}
Glucose + Arabinose	Butanol	0.27 ± 0.003^{c}	0.23 ± 0.003^{c}	0.29 ± 0.003^{c}	0.25 ± 0.003^{d}	0.36 ± 0.005^{d}	0.28 ± 0.001^{d}
	ABE	0.42 ± 0.002^{c}	0.33 ± 0.003^{c}	0.45 ± 0.005^{d}	0.39 ± 0.005^{d}	0.54 ± 0.002^{d}	0.42 ± 0.005^{d}
Glucose + Xylose	Butanol	0.27 ± 0.005^{e}	0.21 ± 0.002^{e}	0.27 ± 0.004^{e}	0.22 ± 0.004^{e}	0.31 ± 0.002^{f}	0.24 ± 0.004^{f}
	ABE	0.41 ± 0.003^{e}	0.33 ± 0.001^{e}	0.44 ± 0.004^{f}	0.36 ± 0.003^{f}	0.48 ± 0.003^{f}	0.37 ± 0.002^{f}
Arabinose	Butanol	0.26 ± 0.003^{g}	0.27 ± 0.003^{g}	0.27 ± 0.003^{g}	0.29 ± 0.003^{g}	0.35 ± 0.006^{h}	0.31 ± 0.003^{g}
	ABE	0.40 ± 0.005^{g}	0.33 ± 0.004^{g}	0.46 ± 0.001^{h}	0.38 ± 0.004^{h}	0.51 ± 0.005^{h}	0.46 ± 0.003^{h}
Xylose	Butanol	0.30 ± 0.006^{i}	0.20 ± 0.004^{i}	0.29 ± 0.001^{i}	0.20 ± 0.004^{i}	0.31 ± 0.003^{i}	0.21 ± 0.005^{i}
	ABE	0.45 ± 0.007^{i}	0.30 ± 0.003	0.44 ± 0.001^{i}	0.30 ± 0.004^{i}	0.47 ± 0.002^{i}	0.32 ± 0.006^{i}

Errors (±) denote standard deviations (n = 3). All deviations are ≤ 0.007. Tukey's pairwise comparison was applied to the means of butanol and ABE yields and productivities observed in cultures of *C. beijerinckii*_pMTL500E, _M1GS^{CcpA}, and _M1GS^{DisA}. Means for _M1GS^{CcpA}, and _M1GS^{DisA} were compared to _ pMTL500E. Means for _M1GS^{CcpA}, and _M1GS^{DisA} with the same alphabet superscripts as _pMTL500E do not vary significantly from _pMTL500E, while those with different superscripts do ($p \leq 0.05$). Comparisons were made for the different media: glucose$^{\mathbf{a,b}}$; glucose+arabinose$^{\mathbf{c,d}}$; glucose+xylos$^{\mathbf{e,f}}$; arabinose$^{\mathbf{g,h}}$; xylose$^{\mathbf{i,j}}$.

Supplementary Table 3. Residual sugars in cultures of the three *C. beijerinckii* strains after fermentation on various sugars for 60 h.

**Substrate**	***C. beijerinckii*_pMTL500E**		***C. beijerinckii*_M1GS^{CcpA}**		***C. beijerinckii*_M1GS^{DisA}**	
Glucose	21.0 ± 0.7^{a}		25.7 ± 0.7^{b}		24.2 ± 1.0^{b}	
Glucose + arabinose	Glucose	Arabinose	Glucose	Arabinose	Glucose	Arabinose
	13.7 ± 0.33^{c}	15.6 ± 0.08^{c}	13.0 ± 0.04^{c}	15.1 ± 0.01^{c}	12.1 ± 0.15^{d}	14.8 ± 0.06^{c}
Glucose + xylose	Glucose	Xylose	Glucose	Xylose	Glucose	Xylose
	13.8 ± 0.74^{e}	15.7 ± 0.25^{e}	15.0 ± 0.05^{f}	13.0 ± 0.14^{f}	13.8 ± 0.05^{e}	13.1 ± 0.07^{f}
Arabinose	28.0 ± 0.65^{g}		27.6 ± 0.71^{g}		25.8 ± 0.13^{h}	
Xylose	27.8 ± 0.47^{i}		27.31 ± 0.811^{i}		26.6 ± 0.36^{j}	

Errors (±) denote standard deviations of the mean (n = 3). Tukey's pairwise comparison was applied to the means of residual glucose, arabinose and xylose concentrations observed in cultures of *C. beijerinckii*_pMTL500E, _M1GS^{CcpA}, and _M1GS^{DisA}. Means with same letters as those used for *C. beijerinckii*_pMTL500E in the different media (glucose$^{\mathbf{a,b}}$; glucose + arabinose$^{\mathbf{c,d}}$; glucose + xylose$^{\mathbf{e,f}}$; arabinose$^{\mathbf{g,h}}$; xylose$^{\mathbf{i,j}}$) do not vary significantly from_pMTL500E, while those with different letters vary significantly ($p \leq 0.05$).

Supplementary Table 4. Annotation of alanine racemase genes in *C. beijerinckii* NCIMB 8052 that are similar to Cbei_0047 (CcpA/alanine racemase).

Gene	Annotation[#]	Percent similarity at the amino acid level to CcpA in	
		*C. beijerinckii**	*C. acetobutylicum***
Cbei_0047	**Alanine racemase/CcpA** – sugar-binding domain; Ligand binding domain of the LacI tanscriptional regulator family; Helix-turn-helix (HTH) DNA binding domain of the LacI family of transcriptional regulators	100	63
Cbei_0731	**Alanine racemase** – sugar-binding domain; Ligand binding domain of the LacI tanscriptional regulator family; Helix-turn-helix (HTH) DNA binding domain of the LacI family of transcriptional regulators	39	42
Cbei_0863	**Alanine racemase** – sugar-binding domain; Ligand binding domain of the LacI tanscriptional regulator family; Helix-turn-helix (HTH) DNA binding domain of the LacI family of transcriptional regulators	36	34
Cbei_4148	**Alanine racemase** – sugar-binding domain; Ligand binding domain of the LacI tanscriptional regulator family; Helix-turn-helix (HTH) DNA binding domain of the LacI family of transcriptional regulators	35	34
Cbei_2457	**Alanine racemase** – sugar-binding domain; Ligand binding domain of the LacI tanscriptional regulator family; Helix-turn-helix (HTH) DNA binding domain of the LacI family of transcriptional regulators	35	34
Cbei_1831	**Alanine racemase** – sugar-binding domain; Ligand binding domain of the LacI tanscriptional regulator family; Helix-turn-helix (HTH) DNA binding domain of the LacI family of transcriptional regulators	33	34
Cbei_4422	**Alanine racemase** – sugar-binding domain; Ligand binding domain of the LacI tanscriptional regulator family; Helix-turn-helix (HTH) DNA binding domain of the LacI family of transcriptional regulators	30	32
Cbei_0234	**Alanine racemase** – ycjW transcription regulator; Ligand-binding domain of uncharacterized transcription regulator ycjW, a member of the LacI-GalR family repressors; Helix-turn-helix (HTH) DNA binding domain of the LacI family of transcriptional regulators	30	30

[#]Information on gene annotation was obtained from the National Center for Biotechnology Information (NCBI) database. **C. beijerinckii* NCIMB 8052; ***C. acetobutylicum* ATCC 824
